# Acquired resistance to the second-generation androgen receptor antagonist enzalutamide in castration-resistant prostate cancer

**DOI:** 10.18632/oncotarget.8456

**Published:** 2016-03-29

**Authors:** Steven Kregel, James L. Chen, Westin Tom, Venkatesh Krishnan, Jacob Kach, Hannah Brechka, Tim B. Fessenden, Masis Isikbay, Gladell P. Paner, Russell Z. Szmulewitz, Donald J. Vander Griend

**Affiliations:** ^1^ Committee on Cancer Biology, The University of Chicago, Chicago, IL, USA; ^2^ Department of Biomedical Informatics and Internal Medicine, The Ohio State University, Columbus, OH, USA; ^3^ Department of Medicine, Section of Hematology/Oncology, The University of Chicago, Chicago, IL, USA; ^4^ Department of Pathology, The University of Chicago, Chicago, IL, USA; ^5^ Department of Surgery, Section of Urology, The University of Chicago, Chicago, IL, USA

**Keywords:** androgen receptor, prostate cancer, castration-resistance, enzalutamide

## Abstract

Enzalutamide (MDV3100) is a second generation Androgen Receptor (AR) antagonist with proven efficacy in the treatment of castration resistant prostate cancer (CRPC). The majority of treated patients, however, develop resistance and disease progression and there is a critical need to identify novel targetable pathways mediating resistance. The purpose of this study was to develop and extensively characterize a series of enzalutamide-resistant prostate cancer cell lines. Four genetically distinct AR-positive and AR-pathway dependent prostate cancer cell lines (CWR-R1, LAPC-4, LNCaP, VCaP) were made resistant to enzalutamide by long-term culture (> 6 months) in enzalutamide. Extensive characterization of these lines documented divergent *in vitro* growth characteristics and AR pathway modulation. Enzalutamide-resistant LNCaP and CWR-R1 cells, but not LAPC-4 and VCAP cells, demonstrated increased castration-resistant and metastatic growth *in vivo*. Global gene expression analyses between short-term enzalutamide treated vs. enzalutamide-resistant cells identified both AR pathway and non-AR pathway associated changes that were restored upon acquisition of enzalutamide resistance. Further analyses revealed very few common gene expression changes between the four resistant cell lines. Thus, while AR-mediated pathways contribute in part to enzalutamide resistance, an unbiased approach across several cell lines demonstrates a greater contribution toward resistance via pleiotropic, non-AR mediated mechanisms.

## INTRODUCTION

Since the 1940′s the standard of care for the treatment of advanced prostate cancer has focused on the inhibition of the Androgen Receptor (AR) signaling axis. In the normal prostate AR is necessary for the function, survival, and differentiation of prostatic tissue [1–3]. In contrast, during carcinogenesis the function of AR signaling is altered from tumor suppressive to tumor promoting [2, 4, 5]. Surgical or chemical castration targeting the androgen receptor signaling axis has been the mainstay of prostate cancer treatment since the landmark study by Charles Huggins and Clarence Hodges in 1941 [6]. Unfortunately, the effects of castration are temporary and after treatment prostate cancer will again progress to what is termed castration-resistance [7]. Although castration-resistant prostate cancer (CRPC) is by definition no longer controlled by testosterone suppression alone, the clinical development of second-generation AR antagonists, including enzalutamide, has demonstrated that the AR remains a critical oncogene in CRPC. Response to enzalutamide, however, is temporary and overall survival in CRPC patients is only modestly increased [8, 9].

The current clinical paradigm for the treatment of prostate cancer, even in the castration-resistant state, remains focused on the blockade of AR signalling. Whether prostate cancer (PC) cells adapt to enzalutamide through alterations to AR directly (e.g. mutation or splice-variation), or whether there are distinct non-AR adaptive mechanisms of resistance is a fundamental question in understanding the development of enzalutamide resistance. Most of the work studying AR antagonist resistance has focused on activating mutations on the AR ligand binding domain (LBD) and splice variants, particularly the constitutively active AR- variants that lack the ligand binding domain [10–16]. Such AR modifications explain only a fraction of clinical resistance to potent AR-targeted CRPC therapy [12, 15], and the contribution of non-AR mediated resistance mechanisms remains poorly understood.

Previous data has supported a role for alternate mechanisms of resistance that may contribute to enzalutamide resistance. For example, expression and activity of the Glucocorticoid Receptor (GR) and the pluripotent stem cell transcription factor Sox2 have been shown by our group and others to promote enzalutamide resistance [17–22]. We hypothesized that there are other, similarly unifying, and targetable pathways linked to resistance. The purpose of this study was to develop and extensively characterize a series of enzalutamide-resistant prostate cancer cells to enable future mechanistic studies. To model the innate heterogeneity among patients, we cultured four distinct AR-positive prostate cancer cell lines continuously in enzalutamide for at least six months upon which they were considered enzalutamide resistant (Enz^R^). We compared differences in proliferation, viability, resistance to docetaxel, and *in vivo* growth to the parental and cells treated short-term (48 hours) with enzalutamide. We also characterized changes in gene expression longitudinally during the evolution of resistance. These studies revealed heterogeneous characteristics acquired by each cell line as well as key differences in AR pathway adaptation. Our data shows that although AR-mediated pathways contribute to enzalutamide resistance, an unbiased approach across several cell lines indicates that there may be a significant contribution from pleiotropic, non-AR mediated mechanisms.

## RESULTS

### *In vitro* Enz^R^ cell line characterization

We chose four genetically distinct PC cell lines to chronically treat with 10 μM enzalutamide for at least six months to model disease progression during treatment. The four lines were chosen for their unique and clinically relevant AR protein status. CWR-R1 cells were derived from the transplantable castration resistant xenograft, CWR22, which was initially derived from a primary tumor of a patient with bone metastases [23, 24]. These cells harbor a histidine to tyrosine at residue 874 mutation in the AR ligand binding domain (LBD), which enables broadened ligand responsiveness and influences the binding of coactivator proteins [25, 26]. CWR-R1 cells also maintain stable expression of many constitutively active AR splice variants that lack the LBD, including AR-V7 [27]. LNCaP cells, which were derived from a patient lymph node metastasis have a mutated AR containing the threonine to alanine mutation at amino acid 877; this mutation has been found in both naïve and castration resistant prostate cancer patient samples [25, 28]. The mutation within the LBD also confers broadened ligand responsiveness and activation by a variety of hydrophobic biomolecules [25]. LAPC-4 cells were also derived from a patient lymph node metastasis, but they express wild-type AR and are reliant on exogenous androgen to thrive in culture [29]. Finally, VCaP cells were derived from a human vertebral metastasis xenograft and have amplified expression of the AR, the most common mechanism of castration resistance in patient samples [30, 31]. VCaP cells also express detectable levels of AR-V7, and the common AR-driven *TMPRSS2-ERG* gene fusion [30, 31].

**Figure 1:**
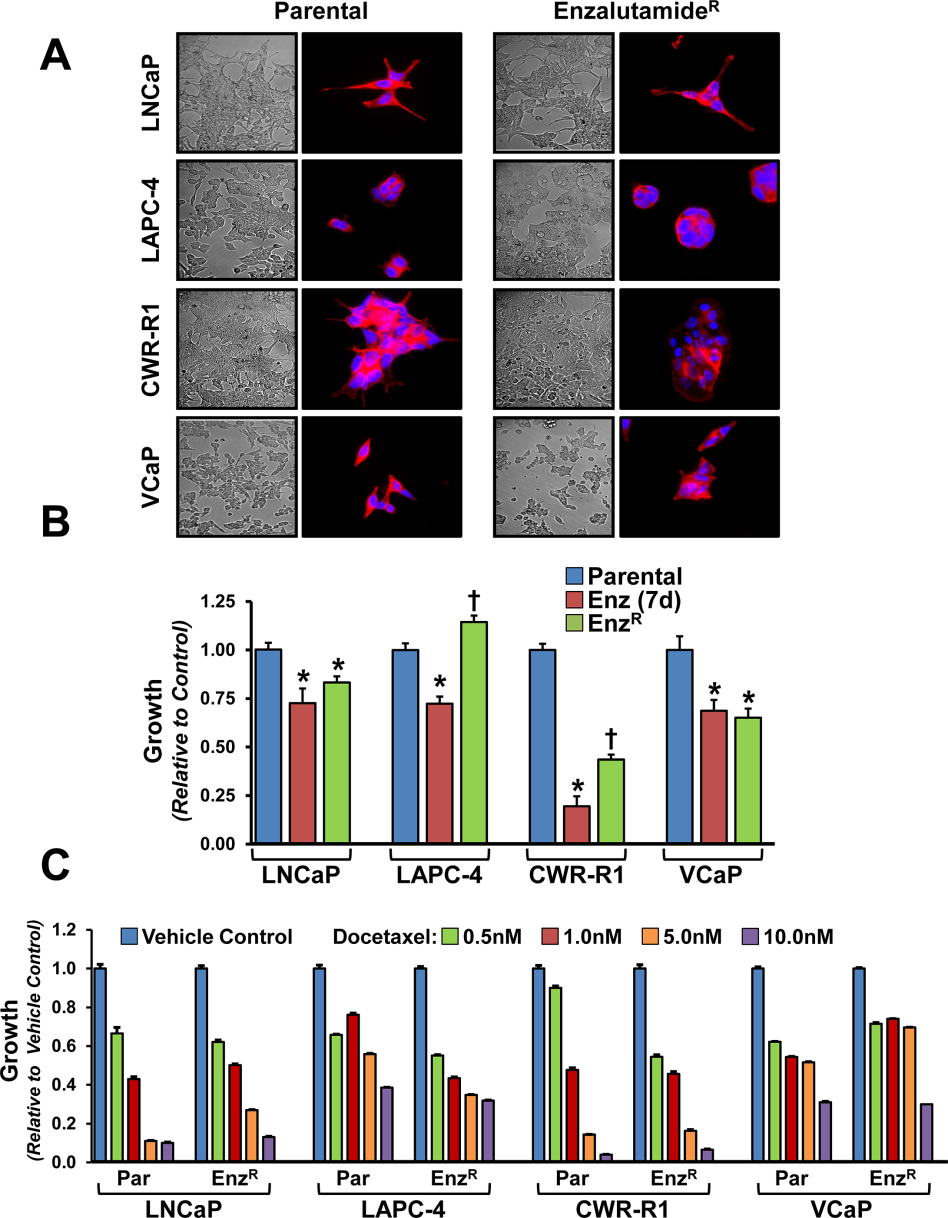
Morphology, growth rates and sensitivity to docetaxel of enzalutamide resistant and parental cell lines (**A**) Phase contrast and Fluorescent images taken at 60x of Parental and respective enzalutamide resistant (Enz^R^) cell lines. Rhodamine conjugated Phalloidin (Red) which stains the F-actin cytoskeleton [50] is used to outline cellular morphology, and DAPI (blue) is a DNA stain used to outline the nuclei [51]. Cross signifies statistical significance to both parental and short-term treated cells, *indicated *p*-value < 0.05. (**B**) Relative growth rates of Parental, Enz^R^ (grown in 10 μM enzalutamide) and cells acutely treated (7 days) with 10 μM enzalutamide measured by MTT. (**C**) Relative growth rates of Parental and Enz^R^ (grown in 10 μM enzalutamide) cells grown in increasing concentrations (0.5, 1, 5 and 10 nM) of the microtubule stabilization chemotherapeutic agent, Docetaxel.

All four prostate cancer cell lines were treated continuously with enzalutamide; the surviving and proliferating resistant cells were pooled, maintained, and subsequently compared to their matched parental counterparts (termed Enz^R^ cells). These resistant cell lines were characterized and compared to their matched parental cell lines, as well as parental cells treated for 2–7 days in enzalutamide. After selection in enzalutamide, the Enz^R^ cells displayed no overt changes in morphology when compared to their matched parental cell lines (Figure 1A). As anticipated, in all four cell lines short-term enzalutamide-treatment over seven days led to a statistically significant decrease in cell number (Figure 1B). Interestingly, even the castration-resistant CWR-R1 cells, which contain the AR-V7 splice variant, demonstrate a statistically significant growth inhibition in response to short-term enzalutamide treatment compared to parental (Figure 1B). Enz^R^ cells displayed heterogeneous growth characteristics when compared to their parental cell lines under standard growth conditions or over seven days of enzalutamide treatment. LNCaP-Enz^R^ and VCaP-Enz^R^ cells maintain suppressed growth on enzalutamide indistinguishable from short-term treated cells. However, over a seven day time period there were more CWR-R1-Enz^R^ and LAPC-4-Enz^R^ cells compared to their respective short-term enzalutamide treated cells (Figure 1B). Compared to their parental cells, CWR-R1-Enz^R^ had fewer cells after seven days; whereas in LAPC-4 line, the most cells after seven days were in the LAPC-4- Enz^R^ line (Figure 1B). For the LNCaP-Enz^R^ and CWR-R1-Enz^R^ cells, since there were fewer cells compared to parental, we surmised that there may be some degree of ongoing cell death with enzalutamide treatment despite resistance. To test this, we measured propidium-iodide uptake in steady-state parental vs. Enz^R^ cultures (the Enz^R^ cells were maintained in enzalutamide). This showed decreased cellular viability within the CWR-R1-Enz^R^ cell line (13.2 ± 0.97% dead in parental vs. 48.85 ± 3.24% dead in Enz^R^ cells; *p* = 0.014), and the LNCaP-Enz^R^ cells (4.12 ± 0.22% dead in parental vs. 7.13 ± 0.71% dead in Enz^R^ cells; *p* = 0.028). In the LAPC-4-Enz^R^ cells, which had more cells compared to parental (Figure 1B), there was no difference in cell death via PI uptake (20.64 ± 1.72 dead in parental vs. 19.4 ± 0.87% dead in Enz^R^ cells; *p* = 0.379). Thus, the cell lines with decreased cellular growth over time can be attributed to continued cell death despite enzalutamide resistance. To understand to what extent Enz^R^ cells proliferation was impacted by enzalutamide-resistance, we withdrew enzalutamide from our Enz^R^ cells. Cessation of enzalutamide treatment had little effect on the subsequent growth rates of the Enz^R^ cells, except in the LNCaP-Enz^R^ cells which responded to enzalutamide withdrawal with an increase in growth in comparison to when enzalutamide treatment is maintained (Supplementary Figure S1). These data show diversity in cellular growth and death rate upon acquisition of enzalutamide resistance and support a model in which there may be heterogeneous molecular mechanisms underlying this resistance.

We also investigated resistance to docetaxel, a standard chemotherapy used to treat CRPC, with the hypothesis that enzalutamide resistance may confer multi-drug resistance. In all four cell lines, Enz^R^ cells continued to be sensitive to docetaxel-induced cell death (Figure 1C); slight variations, however, in the magnitude of cell death relative to the parental line were observed. These differences are likely due differences in cell proliferation and their sensitivities to anti-mitotic, taxane chemotherapeutics [32]. Thus, there is no evidence of cross-resistance to docetaxel in our Enz^R^ lines.

### Castration-Resistant *in vivo* growth of Enz^R^ cells

**Figure 2:**
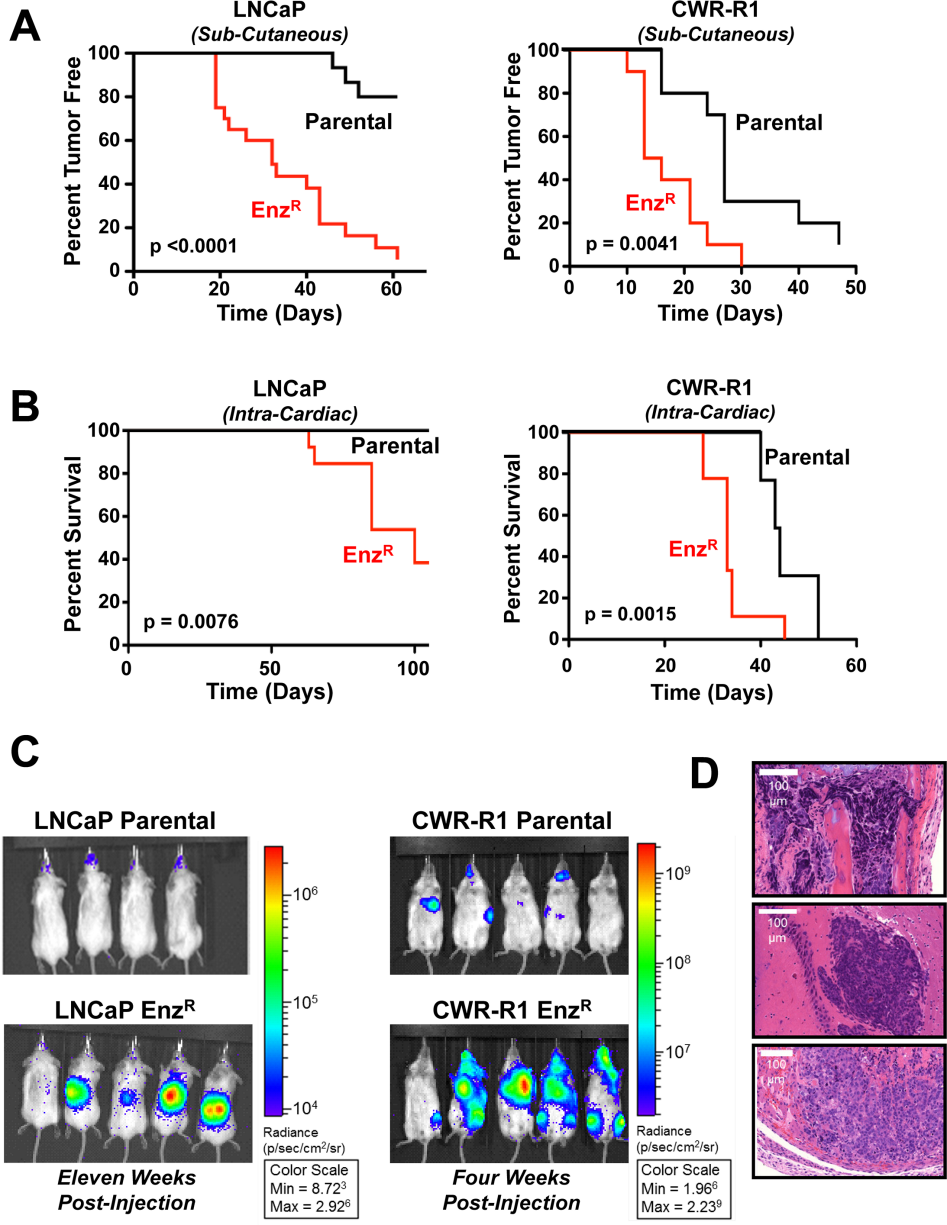
Enzalutamide resistant LNCaP and CWR-R1 cells display increased castration resistant tumor take, and increased metastatic colonization to bone (**A**) Kaplan-Meier survival curves illustrating time to tumor take (measured as > 100 mm^3^) in a castrated male nude mice. Parental and Enz^R^cells were injected subcutaneously on the flanks of castrated nude mice: LNCaP Parental (*N* = 20) vs LNCaP Enz^R^ (*N* = 20) (Left Panel; *p* < 0.0001); CWR-R1 Parental (*N* = 10) vs. CWR-R1 Enz^R^ (*N* = 10) (Right Panel; *p* = 0.0041). (**B**) Kaplan-Meier survival curves illustrating overall survival of mice post-intracardiac injection to the veterinarian approved endpoint. LNCaP parental (*N* = 8) vs. LNCaP Enz^R^ (*N* = 13) cells (Left Panel; *p* = 0.0076); CWR-R1 parental (*N* = 12) vs. CWR-R1 Enz^R^ (*N* = 9) cells (Right Panel; *p* = 0.0015). (**C**) Representative images of *in vivo* bioluminescent imaging of the metastatic colonization of the LNCaP Parental and Enz^R^ intracardiac injected mice (Left Panel, Dorsal side shown), and CWR-R1 Parental and Enz^R^ intracardiac injected mice (Right Panel, Ventral side shown). (**D**) Representative H & E images (20× magnification) of histological sections of metastases obtained from CWR-R1 Enz^R^ injected mice. Metastases were confirmed by a pathologist in the Bone (Top image, Tibia), Brain (Middle image) and Adrenal glands (Bottom image) (additional images in Supplementary Figures S2 and S3). Scale bar represents 100 μm.

Since the Enz^R^ prostate cancer cell lines continue to grow *in vitro* even in the presence of enzalutamide, we hypothesized that they would display castration-resistant growth *in vivo*. To test this we analyzed the growth of the different cell lines *in vivo* after inoculation into castrated male athymic nude mice. Interestingly, both VCaP-Enz^R^ and LAPC-4-Enz^R^ cells displayed very low and not significantly increased castration-resistant tumor initiation (measured as time to 100 mm^3^ tumor growth) compared to parental [1/18 and 1/18 for VCaP and VCaP Enz^R^ respectively, and 0/30 and 0/34 for LAPC-4 and LAPC-4-Enz^R^, respectively (tumors formed/injection sites) by 100 days post-inoculation]. However, the LNCaP-Enz^R^ cells demonstrated robust castration-resistance, whereby tumor initiation of the LNCaP-Enz^R^ cells was 95% compared to only 15% in parental cell lines, and median tumor initiation was accelerated by over one month (Figure 2A). Similarly, for the aggressive and *de novo* castration resistant CWR-R1 cell line, time to castration-resistant tumor initiation was accelerated an average of 14 days in the Enz^R^ cells compared to parental (Figure 2A).

We further examined this striking phenotype by investigating castration-resistant metastatic colonization of the LNCaP-Enz^R^ and CWR-R1-Enz^R^ cells compared to their respective parental line. Intracardiac (IC) injection of luciferase-expressing cells into castrated male SCID mice followed by bioluminescence imaging showed significantly increased metastatic colonization of Enz^R^ cells (Figure 2B and 2C). Overall survival of mice post-intracardiac injection was significantly decreased when using Enz^R^ resistant lines compared to parental lines. Median mortality was increased by eleven days in CWR-R1-Enz^R^ lines, and 60% of mice were deceased by 100 days in LNCaP-Enz^R^ tumor-bearing mice, with no mortality seen using the parental cell line (Figure 2B). This decrease in metastatic latency mirrors subcutaneous castration-resistant tumor initiation in these lines. Metastatic colonization to many clinically-relevant organ sites was validated histologically and included the bone [long bones (tibia, femur, humerus), vertebrae, and skull (maxilla and mandible)], brain and nerves (cortex, cerebellum, olfactory bulb, dorsal root ganglion), and adrenal glands (Figure 2D, Supplementary Figures S2 and S3). In sum, initial characterization documents variable *in vitro* and *in vivo* growth patterns of Enz^R^ cells, and implies mechanistic diversity in acquisition of enzalutamide resistance between the four lines.

### Analysis of AR status in Enz^R^ lines

Since the AR has been a focal point in enzalutamide resistance investigation to date, and given our variable *in vitro* and *in vivo* findings, we next sought to interrogate changes in AR mRNA and protein during the development of resistance. Sanger sequencing of pooled cDNA of both parental and Enz^R^ cells demonstrated that the AR LBD acquired no new mutations in any of the resistant lines (CWR-R1 still contained the histidine to tyrosine at residue 874, LNCaP still contained the Threonine to Alanine mutation at amino acid 877, and LAPC-4 and VCaP maintained wild type AR) [14]. Furthermore, there was no detectable testosterone found in tissue culture conditioned media in all cell lines. These data confirm that the cells did not develop a dominant clone containing the previously described Phenylalanine to Leucine mutation at 876 which allows enzalutamide to act as an AR agonist, nor did they increase endogenous ligand by increased testosterone biosynthesis [33].

**Figure 3:**
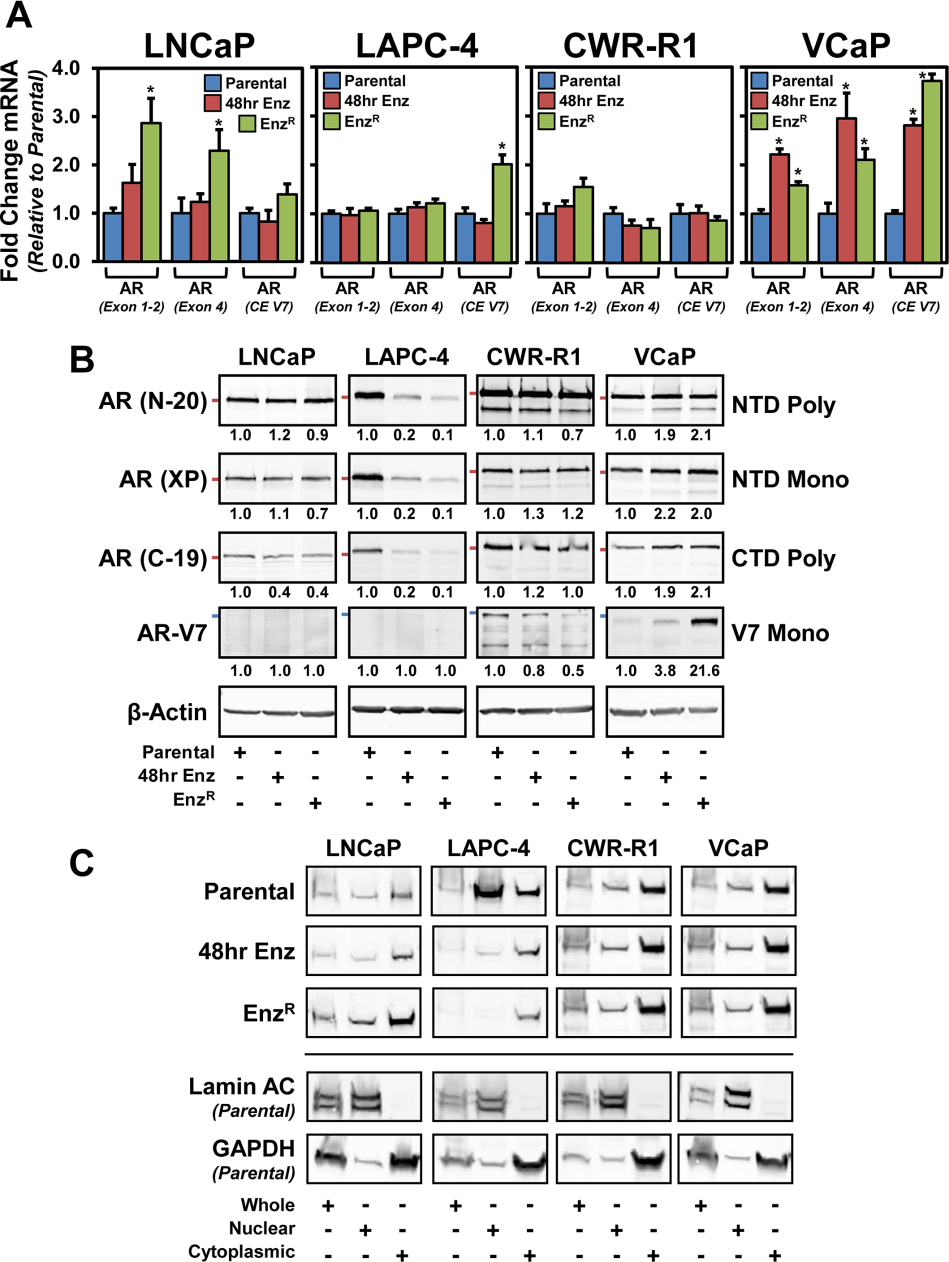
The status of the androgen receptor (AR) in parental and respective enzlutamide-resistant (Enz^R^) cell lines (**A**) Analysis of AR mRNA expression from Parental, Enz^R^ (grown in 10 μM enzalutamide) and cells acutely treated (48 hours) with 10 μM enzalutamide to identify splice variants and truncations. One set of primers spans the junction between exons 1 and 2 to identify total AR expression, another is designed to identify most full length AR transcripts, and finally primers were designed to AR-V7 within cryptic exon (CE) 3b, to gain a broader sense of total, full length and splice variant expression. Expression was normalized to GAPDH using the ΔΔCT method. (**B**) Western blot analysis to determine AR protein levels. Whole cell lysates from Parental, Enz^R^ (grown in 10 μM enzalutamide) and cells acutely treated (48 hours) with 10 μM enzalutamide. We investigated using three different AR antibodies: polyclonal targeting the N-terminus (N-20), monocolonal targeting the N-terminus (XP) and polyclonal targeting the C-terminus (C-19), and AR splice variant V7 expression (precision antibody). β-Actin was used as a loading control. Red Bars demarcate 100kDA molecular weight (AR N-20, XP, and C-19); Blue bars demarcate 75 kDA molecular weight (AR-V7). (**C**) Nuclear localization of AR protein in Enz^R^ cells. Western blot analysis of AR (N-20) from Parental, Enz^R^(grown in 10 μM enzalutamide) and cells acutely treated (48 hours) with 10 μM enzalutamide in cells that were biochemically fractionated to isolate Nuclear and Cytosolic compartments, as well as whole cell lysates. Lamin A/C served as a nuclear control, and GAPDH served as a cytosolic marker. Each lane represents 100,000 cells worth of lysate.

We next investigated the status of androgen receptor expression in the Enz^R^ cells. First, we quantified *AR* mRNA expression using custom primers designed to different regions of the AR transcript. One set of primers span the junction between exons 1 and 2 to identify total *AR* expression, another is designed to identify full length AR transcripts spanning exon 4, and a third primer set specifically detects *AR-V7* within cryptic exon 3b. In LNCaP cells, full-length *AR* mRNA was upregulated in the Enz^R^ cells when compared to parental and cells acutely (short-term 48 hour) treated with enzalutamide (Figure 3A). In LAPC-4 cells, *AR-V7* mRNA was increased (2 fold +/− 0.189) in LAPC-4-Enz^R^ cells, and all other expression was unchanged (Figure 3A). Upon short and long term enzalutamide exposure, VCaP cells increased full-length *AR* as well as *AR-V7* mRNA; no significant change in *AR* expression of any kind was observed within CWR-R1 cells (Figure 3A).

We then tested whether these patterns in AR mRNA were maintained at the protein level (Figure 3B). In the LNCaP and CWR-R1 cells, full-length AR was unchanged under both short term (48 hour) enzalutamide treatment and in the Enz^R^cells, whereas in the VCaP lines enzalutamide treatment lead to increased AR expression as was observed at the mRNA level (Figure 3B). In contrast, full-length AR in LAPC-4 cells was decreased under short-term enzalutamide treatment and was further decreased in the Enz^R^ cells (Figure 3B). We investigated AR-V7 expression using an antibody specific to the unique C-terminal peptide expressed from the cryptic exon 3b not present in full-length AR [34]. These data document that AR-V7 protein expression is undetectable in LNCaP and LAPC-4 cells under all conditions (Figure 3B). Interestingly, in the cell lines that express AR-V7 protein under normal growth conditions, CWR-R1 and VCaP, there are contrasting changes in AR-V7 protein expression with enzalutamide treatment. AR-V7 *decreased* in the CWR-R1-Enz^R^ cells, whereas there was a marked *increase* in the AR-V7 protein expression in VCaP-Enz^R^ cells. We also investigated AR nuclear localization as a surrogate for AR activation with the hypothesis that Enz^R^ cell lines that maintain or increase AR (full-length or V7) expression would have stable or increased nuclear localization of AR. Through biochemical nuclear/cytosolic fractionation, we saw a predominance of cytoplasmic AR subsequent to short and long term enzalutamide treatment (Figure 3C). There was no increase in nuclear localization of the AR in the Enz^R^ cells compared to short term treatment, except in LNCaP where cytoplasmic and nuclear AR are both increased (Figure 3C). Moreover, in the CWR-R1 and VCAP lines, which express the AR-V7 splice variant, there is sustained nuclear AR localization throughout enzalutamide treatment (Figure 3C). These data illustrate that there is no uniform pattern of AR expression and localization subsequent to enzalutamide treatment and resistance. Three of the four lines, however, maintain detectable nuclear AR, suggesting that there may be maintenance of AR signaling upon acquisition of enzalutamide resistance.

### AR target gene expression and signaling are altered and heterogeneous amongst Enz^R^ lines

**Figure 4:**
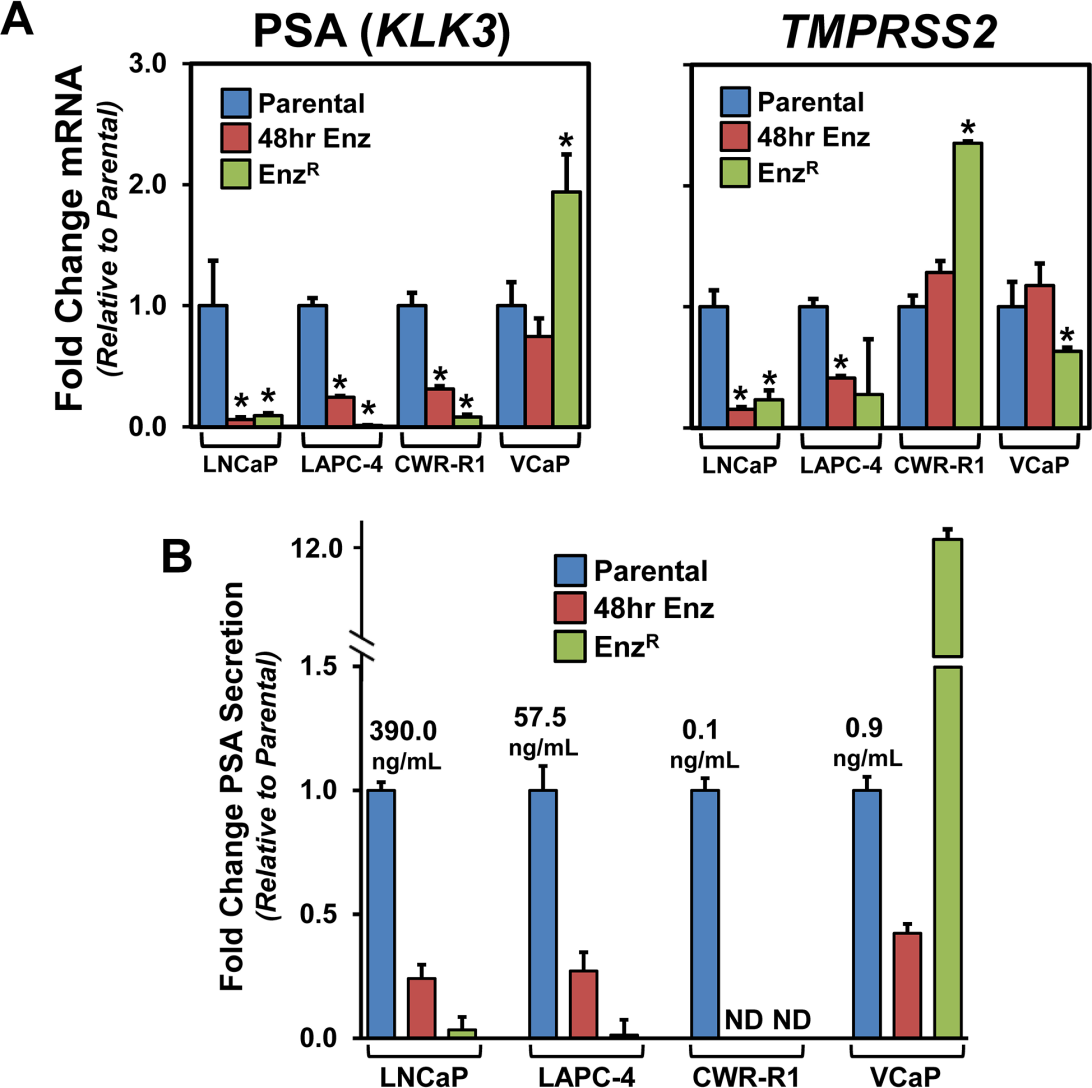
The expression of the AR target genes PSA and TMPRSS2 and the status of AR signaling in the Enz^R^ cell lines (**A**) RNA analyses of canonical AR-target genes, Prostate Specific Antigen (PSA), or *KLK3* [35], and *TMPRSS2* (Transmembrane protease serine 2) [36] using custom designed primers and cDNA from Parental, Enz^R^ (grown in 10 μM enzalutamide) and cells acutely treated (48 hours) with 10 μM enzalutamide. (**B**) PSA protein expression of conditioned media from Parental, Enz^R^ (grown in 10 μM enzalutamide) and cells acutely treated (48 hours) with 10 μM enzalutamide grown for 48 hours.

We next tested whether there would be concurrent restoration of AR pathway signaling shared between the Enz^R^ cell lines. This restoration could be through ligand-independent AR activation/signaling or activation of AR- target genes through alternative pathways [1, 10, 19, 20]. Thus, we tested the expression of specific downstream AR-pathway target genes Prostate Specific Antigen (PSA or *KLK3*) [35], and *TMPRSS2* (Transmembrane protease serine 2) [36]. As anticipated, mRNA expression of these two genes in short-term enzalutamide treated and Enz^R^ cells was decreased in LNCaP and LAPC-4 (Figure 4A). CWR-R1 cells showed decreased *PSA* expression after 48 hours of enzalutamide treatment, and PSA is further decreased in CWR-R1-Enz^R^ cells; however, *TMPRSS2* expression did not change with short term treatment and interestingly increased ~3-fold in the Enz^R^ cells (Figure 4A). In VCaP cells, short-term 48 hour treatment with enzalutamide had no significant effect on *PSA* and *TMPRSS2* expression; however there was a 2-fold increase in *PSA* expression, and a decrease of *TMPRSS2* expression (Figure 4A). Secreted PSA protein levels paralleled the heterogeneous changes in seen in mRNA expression in all cell lines (Figure 4B).

### Analysis of adaptive altered AR-Regulated gene expression in Enz^R^ cells

**Figure 5:**
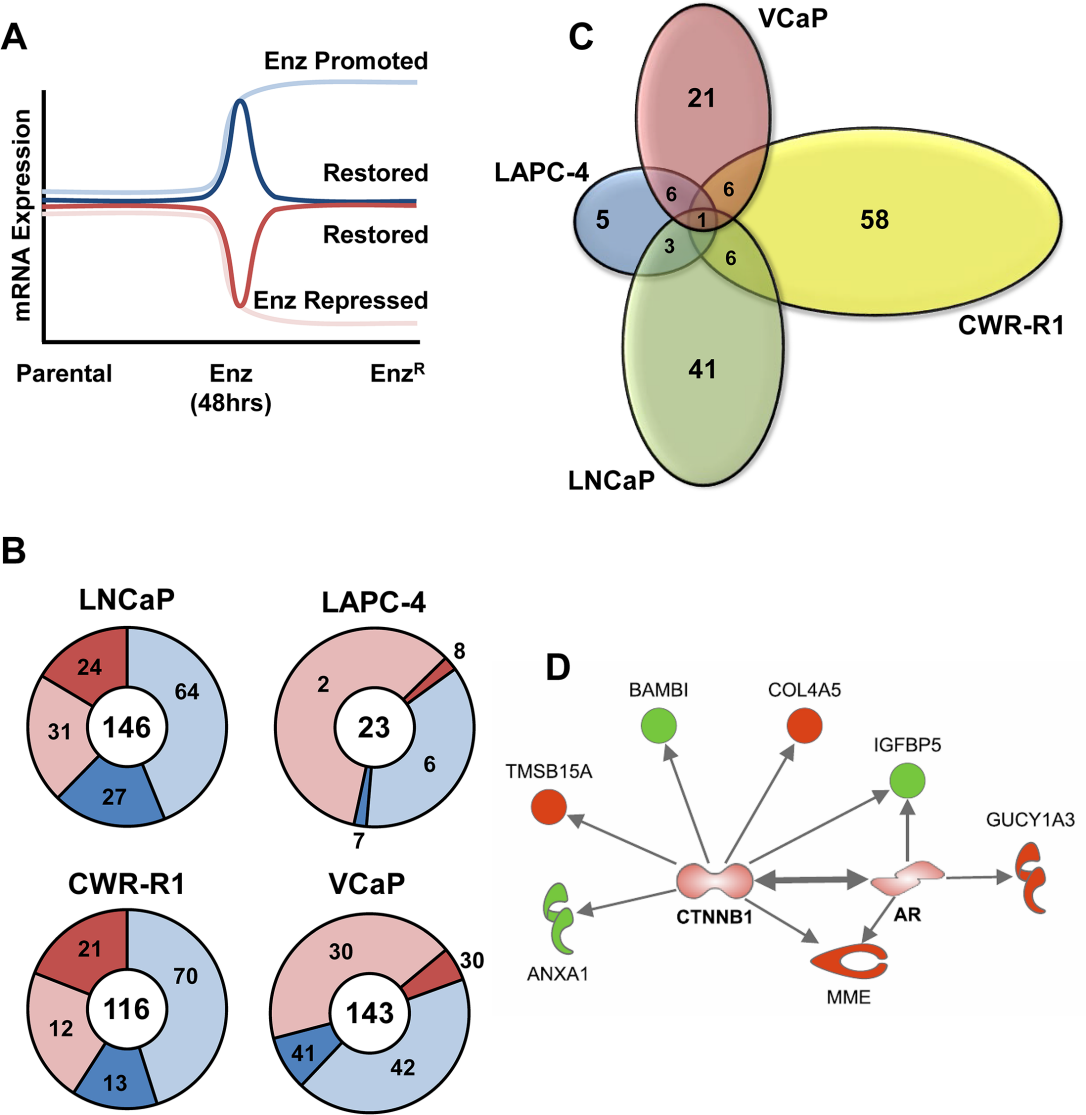
Analysis of adaptive altered AR-Regulated gene expression in Enz^R^ cells (**A**) Schematic illustrating how AR- regulated restored genes were identified. Gene expression analysis to identify AR-pathway promoted and repressed gene expression differences between Parental and cells acutely treated (48 hours) with 10 μM enzalutamide, and which genes were then “restored” in the Enz^R^ cells (grown in 10 μM enzalutamide), or altered back to the expression levels normally found in the Parental lines (Dark Red and Blue Lines). (**B**) Overall numbers of genes promoted or repressed by enzalutamide treatment (light blue, and light red, respectively), then restored to the levels normally found in the respective parental lines (dark blue or dark red, respectively). (**C**) Venn Diagrams illustrating the overlap analysis of the “restored” gene subsets. Only one gene, *TMEFF2* (Transmembrane protein with EGF-like and two follistatin-like domains-2), is a common gene restored across all cell lines. (**D**) Ingenuity Pathway Analysis^®^ (IPA) performed on the “restored” gene subsets that are shared by at least two of the four lines prioritizes both the AR and β-Catenin (*CTNNB1*) pathways (both AR and *CTNNB1* genes not altered by mRNA but imputed by IPA). Proteins outlined in bold red represent genes highly expressed, while genes shown in bold green have lower expression.

Given the strikingly varied, and in some instances divergent, changes in AR expression and pathway activity between all of the different cell lines, we sought to identify adaptive gene expression changes associated with the acquisition of enzalutamide resistance. Schematically, these are genes that change initially with short term enzalutamide treatment and are restored upon enzalutamide resistance (Figure 5A – dark blue and dark red). This restoration could be through ligand-independent AR activation/signaling or activation of AR-target genes through alternative pathways. We took a global approach to test this hypothesis using mRNA gene expression microarray profiling (summarized in Supplementary Table S1). First, we identified all gene expression changes after short term (48 hour) enzalutamide treatment. Within this cohort of genes, we then identified genes that displayed restored expression in the Enz^R^ cell lines (See Supplementary Table S2 for gene lists). As shown in Figure 5B and Supplementary Table S1, of the over 20,000 genes analyzed a very small set of genes are significantly changed with enzalutamide treatment, and a further minority of these genes are restored upon enzalutamide resistance. These restored genes represent a set of candidate genes that may be essential for acquisition of enzalutamide resistance. When comparing genes restored upon enzalutamide resistance, there is strikingly little overlap between all four cell lines (Figure 5C). Only one gene, *TMEFF2* (Transmembrane protein with EGF-like and two follistatin-like domains-2), is a common gene restored across all cell lines. *TMEFF2* is a previously identified AR-target gene, which has been shown to exhibit anti-proliferative effects in prostate cancer cells [37].

We next performed pathway enrichment using Ingenuity Pathway Analysis^®^ (IPA) [38] on restored genes shared by at least two of the four cell lines (Figure 5D and Supplementary Table S4). As expected, AR was a prioritized pathway, and interestingly the other prioritized pathway was the β-Catenin (*CTNNB1*), implicating a role for β-Catenin in mediating enzalutamide resistance (Figure 5D). Overall, these data illustrate the heterogeneity among the four different cell lines in response to chronic treatment of enzalutamide but suggest that β-Catenin signaling may play a key role in mediating enzalutamide resistance. Overall, these data illustrate the heterogeneity among the four different cell lines in response to chronic treatment of enzalutamide and AR-pathway associated genes that confer enzalutamide resistance.

### Analysis of adaptive non-AR-associated gene expression in Enz^R^ cells

**Figure 6:**
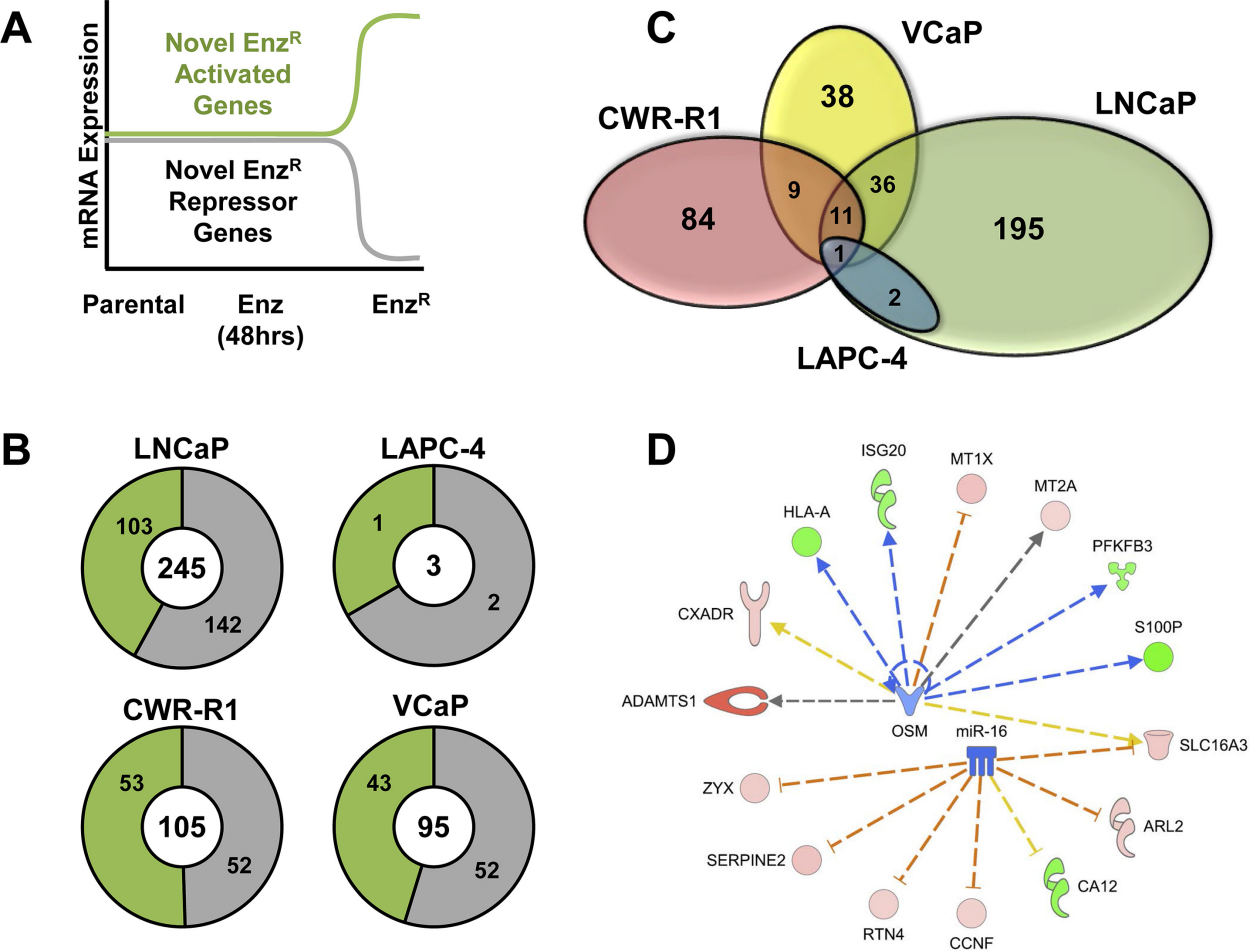
Analysis of adaptive altered non-AR-associated gene expression in Enz^R^ cells (**A**) Schematic illustrating how novel and non-AR-pathway associated genes that promote enzalutamide resistance were identified. Non-AR-pathway associated genes altered in Enz^R^ cells were genes that did not change significantly with short term AR antagonism (48 hrs), but were significantly changed upon acquisition of resistance. The green line represents these novel genes that are *increased* during enzalutamide resistance (Activated), and the gray line represents those genes that are *decreased* during enzalutamide resistance (Repressed). (**B**) Overall numbers of genes Non-AR-Associated genes identified in each cell line (Center Number). Subsets of the numbers in green represent the novel genes that are upregulated during enzalutamide resistance (Activated), and the numbers in gray represent those genes that are downregulated during enzalutamide resistance (Repressed). (**C**) Venn Diagrams illustrating the overlap analysis of the novel non-AR gene subsets in each of the four cell lines. Only one gene is commonly altered restored across all four cancer cell lines: *MT2A* (Metallothionein 2A). (**D**) Ingenuity Pathway Analysis^®^ (IPA) of the non-AR associated resistance genes. IPA was performed on both the genes that shared by at least two of the four lines prioritizes both microRNA 16 (miR-16) and Oncostatin M (*OSM*) pathways. Proteins outlined in red represent genes highly expressed, while genes shown in green have lower expression. Blue arrows represent a positive regulation, which red lines represent a negative regulation, dashed lines represent an indirect interaction.

Non-AR-pathway associated genes altered in Enz^R^ cells were identified as new genes that did not change significantly with short term AR antagonism with enzalutamide but were significantly changed upon acquisition of resistance. A schematic (Figure 6A) illustrates how we identified these novel and non-AR-pathway associated genes that promote enzalutamide resistance. The number of genes that were altered using these analyses range from 245 in LNCaP-Enz^R^ to as few as 3 in LAPC-4-Enz^R^ (Figure 6B, and Supplementary Table S3). Similar to our observations of AR-restored genes (Figure 5,6), there is strikingly little overlap among all four cell lines (Figure 6C). Again, only one gene is commonly altered across all four cancer cell lines: *MT2A* (Metallothionein 2A; Figure 6C). Metallothioneins are small, free-radical scavenging zinc binding proteins that are upregulated in prostate cancer cells resistant to chemotherapy and radiation [39]. Based on these data, Metallothioneins may play a similar role in enzalutamide-resistance.

We then performed pathway enrichment using IPA on non-AR-associated gene sets shared among at least two of the four cell lines (See Supplementary Table S5). Figure 6D illustrates the candidate non-AR pathways activated in the Enz^R^cells: the microRNA 16 (mir16) and Oncostatin M (*OSM*) pathways. While these genes and pathways may serve as novel targets for enzalutamide resistant prostate cancer, our data further highlights the heterogeneity of enzalutamide resistance.

## DISCUSSION

Taken together, these data suggest a variety of different mechanisms and responses utilized by prostate cancer cells to acquire enzalutamide resistance. Beyond the canonical AR-mediated mechanisms of resistance, a broader approach across several cell lines suggests a significant contribution from non-AR mediated mechanisms as well.

The observed heterogeneous changes in how different prostate cancer cell lines acquired enzalutamide resistance raise several clinically significant concerns. First, the increased castration-resistant tumor growth and metastatic ability of two of the four lines demonstrate the potential for accelerating metastatic progression once a patient's tumor has acquired resistance to enzalutamide. This implies that cessation from enzalutamide treatment could result in catastrophic disease progression in certain instances. Further research is needed to identify such patients and understand the mechanisms of increased metastatic progression in response to drug resistance. Second, the heterogeneous responses to enzalutamide between each cell line parallels a lack of uniformity among patients and the need for more precise molecular staging tools to predict patient responses and resistance to enzalutamide. Recent RNA-sequencing of individual CTCs by Miyamoto et al. underscore the vast heterogeneity of mCRPC cells [40]. Third, the maintenance of the AR-pathway within the short term enzalutamide treated and enzalutamide-resistant CWR-R1 and VCaP cells, which contain the V7 AR splice variant lacking the ligand-binding domain, further supports clinical findings that the V7 variant can enable enzalutamide resistance [10, 12]. It should be noted, however, that in some lines acquisition of enzalutamide resistance was not associated with the emergence of the AR-V7 variant. Fourth, the restored expression of a small subset of AR-associated genes in all four Enz^R^ cell lines implies either incomplete inhibition of AR signaling, emergence of AR splice variants driving resistance, or compensatory non-AR activation of key AR-target genes. We have previously reported that glucocorticoid receptor (GR) expression increases in these Enz^R^ lines [19]; GR signaling could explain maintained AR-pathway gene expression.

Outside of the strength of conducting our study across several cell lines representing a range of clinically relevant AR expression, there are some shortcomings and vulnerabilities to our current approach. First, our study was done on pooled resistant clones which allows for the inherent heterogeneity of tumors to be maintained; however an obvious area for future research would be the analyses of multiple resistant clones from the same cell line, or of CRPC patient samples. Individual clones within the population could have masked or overestimated changes in gene expression or phenotypic alterations. Second, the use of microarray is limiting over a technique such as RNA-seq, as our analyses may have missed critical microRNAs, splice variants, and lncRNAs that were not present on the array. Finally, analyses at additional time points may have detected further alterations facilitating enzalutamide resistance, such as early gene expression changes enabling survival several days after the initiation of treatment.

Our bioinformatic analyses prioritized three candidate pathways that may mediate enzalutamide resistance: the β-Catenin, microRNA-16, and Oncostatin M pathways. β-Catenin itself has been shown to enhance AR-pathway activation directly through binding to AR and increasing AR-mediated transcription [41], and previous studies have documented that both β-catenin and TCF4 bind AR in an androgen-dependent manner [41–44]. The oncogenic Wnt/β-Catenin pathway has also been shown to be activated in prostate cancer cells in response to castration and anti-androgen treatment [41, 45]. Furthermore, Miyamoto et al. documented a clear correlation between Wnt signaling and anti-androgen resistance in CTCs derived from patients with advanced, metastatic CRPC; and siRNA knockdown of WNT5A in LNCaP-Enz^R^ cells reduced cell proliferation *in vitro* [40]. The miR16 is a tumor suppressive microRNA that is able to slow cell cycle progression, promote apoptosis and suppress tumorigenicity [46]. Downregulation of miR16, and upregulation of gene targets, could impart adaptive pro-survival and growth mechanism mediating enzalutamide resistance. Finally, the Interleukin-6 family member, Oncostatin M (*OSM*), has previously been shown to promote proliferation and survival in prostate cancer cells [47]. *OSM* signals through the oncogenic JAK-STAT pathway which has known roles in all stages of prostate cancer progression, and members of which are currently being investigated as therapeutic targets in CRPC [48]. Interestingly, our analysis did not prioritize pathways of the negatively regulated AR-target genes GR and Sox2, which we have previously identified as mediating resistance to AR antagonists. Although this may be in part due to our bioinformatics approach and time points selected, these data again illustrate the heterogeneity and variability that characterizes enzalutamide resistance. Further work ongoing with respect to understanding the likely wide ranging and variable mechanisms of resistance is a primary goal of future research.

The Enz^R^ cells described here have the potential to be a valuable research tool for studying CRPC. In particular, the LNCaP-Enz^R^ and the CWR-R1-Enz^R^ cells display castration-resistant metastatic colonization to clinically relevant sites, such as the bone and adrenal glands of mice. The preclinical model presented here provides a crucial investigational tool that the prostate cancer research community needs: a CRPC line that preferentially goes to the bone. Further work needs to be done to identify how these lines acquire the ability to colonize the bone, and the mechanisms by which their outgrowth in the bone could be blocked. Thus, our panel of cell lines can be used to model the heterogeneity in disease seen in clinical contexts and the data presented here suggest novel targets and diagnostic biomarkers that could be used to identify and stratify patients and personalize their care.

## MATERIALS AND METHODS

### Cell lines, growth assays and tissue culture reagents

R1881 was purchased from Sigma-Aldrich (St. Louis, MO), and enzalutamide (MDV3100) was purchased from Selleck Chemicals (Houston, TX), and stored at −20°C in ethanol, and −80°C in DMSO, respectively. All human prostate cancer cell lines were grown as previously described [49], authenticated by DNA Diagnostic Center Medical (DDC, Fairfield, OH), and were routinely screened for the absence of mycoplasma contamination using the ATCC Universal Mycoplasma Detection Kit (Manassas, VA). CWR-R1, LAPC4, and LNCaP cells were generously provided by Dr. John Isaacs at the Johns Hopkins University and have been previously characterized [49]. VCaP cells were attained from ATCC. Luciferase expressing cell lines were created via lentiviral transduction using Promoter-less Luciferase 2 (Promega, Madison, WI) cloned into a pLVX-Hygro lentiviral vector and selected using 300 μg/ml of Hygromycin (Sigma-Aldrich). Cell growth was measured using the Vybrant MTT Cell Proliferation Assay Kit (Invitrogen/Molecular Probes; Eugene, OR) according to manufactures instructions. 2000 cells per well were seeded in poly-D-lysine coated 96 well plates (Becton, Dickinson and Company), then treated with drug the following day. At days three, five, and seven, growth measurements were taken with 4 hour incubation with MTT, and 8 hour development and soluablization with SDS solution at 37°C and measured at 570 nm absorbance. For cell viability, cells were analyzed using the FITC Annexin V Apoptosis Detection Kit with PI (Biolegend; San Diego, CA) and at least 10,000 cells analyzed using an LSR-II flow cytometer (BD Biosciences).

### Generation of enzalutamide-resistant (Enz^R^) cell lines

Greater than 10^^7^ CWR-R1, LAPC4, LNCaP and VCaP cell lines were plated and continuously cultured and maintained in 10 μM enzalutamide for at least 6 months prior towards any experimentation. During this time, > 90% of cells died (assessed visually), and resistant clones were pooled and maintained. Enz^R^ and matched parental cell lines were used within 10 passages of one another, and maintained in culture for approximately the same amount of time. Once resistant, cells were cultured in up to 20 μM enzalutamide without any change in phenotype.

### Western blotting and enzyme-linked immunoassay (ELISA)

Whole-cell lysates collected from cells seeded at 1 × 10^6^ cells per well of a 6 well plate (Becton, Dickinson and Company, Franklin Lakes, New Jersey), were lysed in RIPA-PIC buffer [150 mM sodium chloride, 1.0% Igepal CA-630 (Sigma-Aldrich), 0.5% sodium deoxycholate, 0.1% SDS, 50 mM Tris, pH 8.0, 1× protease inhibitor cocktail (Roche Molecular Biochemicals; Penzberg, Germany)], scraped, and sonicated (Fisher Scientific; Hampton, NH; model FB-120 Sonic Dismembrator). Protein was quantified by BCA assay (Thermo-Fisher Scientific), and 30 μg of protein were loaded per lane. Antibodies used were: anti-AR (C-19, Santa Cruz; Santa Cruz, CA); anti-AR (N-20, Santa Cruz); anti-AR (D6F11 XP^®^, Cell Signaling Technology, Danvers, MA); anti-AR Variant 7 (Precision Antibody, Columbia, MD); anti-Beta Actin (AC-15, Sigma-Aldrich); anti-glyceraldehyde-3-phosphate dehydrogenase [(GAPDH), Cell Signaling Technology]; and anti-Lamin A/C (Clone 14, Millipore, Billerica, MA). Secondary antibodies and Nitrocelluose membranes from Licor (Lincoln, NE) from were used and data captured using a Licor Odyssey system (Lincoln, NE) as previously described [22]. Secreted total Prostate-Specific Antigen (PSA) and Testosterone were measured from conditioned media after 48 hours of growth from cells seeded at 1 × 10^6^ cells per well of a 6 well plate using Roche Elecsys Total Prostate-Specific Antigen (PSA) and Testosterone (T) Assays (Roche Molecular Biochemicals) at the University of Chicago Clinical Chemistry Core.

### Immunofluorescence

Cells grown on ultra-clean glass cover slips (Electron Microscopy Sciences, Hatfield, PA) were washed in Cytoskeletal Buffer [CB: 0.1M 2-(N-morpholino) ethanesulfonic acid (Sigma-Aldrich), 0.03M MgCl_2_ (Sigma-Aldrich), 1.38M KCl (Sigma-Aldrich), pH 6.8], then fixed in 4% Paraformaldehyde (EMS), 1.5% Bovine Serum Albumin (BSA, EMD, Darmstadt, Germany) and 0.5% Triton-X (Sigma-Aldrich) in CB for 15 minutes. Cover slips were then washed 3 times in Phosphate Buffered Solution (PBS, Invitrogen), and incubated with 1:50 AR N-20 (Santa Cruz) antibody and 2 μM Rhodamine conjugated Phalloidin (Invitrogen) in 1.5% BSA (EMD, Darmstadt, Germany) and 0.5% Triton-X (Sigma-Aldrich) in CB overnight at 4°C. Coverslips were then washed again 3 times in PBS, and incubated for 1 hour at 4°C in 1:1000 alexa-488 conjugated goat-anti-rabbit (Invitrogen), 1.5% BSA (EMD) and 0.5% Triton-X (Sigma-Aldrich) in CB. Coverslips were then washed again 3 times in PBS, and mounted onto microscope slides (Fisher Scientific) with Fluormount Aqueous Mounting media (Sigma-Aldrich) with 1:10,000 DAPI solution (Invitrogen). Cells were then imaged using a Nikon Ti-E microscope with a Lumen 200 Pro light source (Prior, Rockland, MA) and an HQ2 cooled CCD camera (Roper Scientific, Sarasota, FL) controlled via Metamorph acquisition software (MDS Analytical Technologies, Sunnyvale, CA), and analyzed with Image J software (National Institutes of Health, Bethesda, MD).

### Quantitative reverse transcription PCR (Q-RT-PCR)

RNA was purified from similar growth conditions described above using the Qiagen RNeasy Mini Kit including the optional DNAse digestion kit (Qiagen, Valencia, CA) and quality tested using an Agilent Bioanalyzer 2100 (Agilent Technologies, Santa Clara, CA). For standard Q-RT-PCR, extracted RNA was converted to cDNA by reverse transcription using SuperScript^®^ III Reverse Transcriptase (Invitrogen). Levels of AR (Exons 1–2, Exon 4), AR-V7, *GAPDH*, *KLK3* [Prostate Specific Antigen (PSA)], and *TMPRSS2* (Transmembrane protease, serine-2) transcripts were quantified using *Power* SYBR^®^ Green Master Mix (Invitrogen) using custom primers (See Supplementary Table S6). Standard curves were used to assess primer efficiency and average change in threshold cycle (ΔCT) values determined for each of the samples relative to endogenous GAPDH levels and compared to vehicle control (ΔΔCT). Experiments were performed in triplicate to determine mean standard error, and student's *t*-tests performed with normalization to control to obtain *p*-values.

### Gene expression analysis and microarray profiling

RNA from samples in biological quadruplicates was purified and quality tested as described in Q-RT-PCR methods. Gene expression profiling was performed using HumanHT-12 v4 Expression BeadChip Kit (Illumina, San Diego, Ca) by the University of Chicago Functional Genomics Core. To minimize batch effect, experiments containing the same cell line were placed on the same chip. Data was then quantile normalized using BRB-Array tools and a 1.5-fold change filter was applied to the samples. Differentially expressed genes were selected using the Significance Analysis of Microarrays (SAM), which performs appropriate tests for multiplicity. Genes that met a 5% false discovery rate (FDR) were retained. Datasets used for this study were deposited into GEO under accession number GSE78201.

### *In vivo* tumor formation

All animal studies were carried out in strict accordance with the recommendations in the Guide for the Care and Use of Laboratory Animals of the National Institutes of Health. The protocol was approved by the University of Chicago Institutional Animal Care and Use Committee (IACUC, protocol numbers 72066 and 72231). All surgery was performed under Ketamine/Xylazine or Isoflurane anesthesia, and all efforts were made to minimize suffering. *In vivo* tumor formation of parental and Enz^R^ cells were conducted via a sub-cutaneous inoculation of one million cells (for LNCaP, VCaP and LAPC-4 lines, 250,000 for CWR-R1 lines) in 4–6 week old male athymic nude mice (Harlan; Indianapolis, IN) using a 75% Matrigel and 25% HBSS solution (BD Biosciences). To measure tumor take in a castrated host, host mice were surgically castrated one week prior to cell inoculation. Intracardiac injections were performed in castrated 4–6 week old C.B.-17 SCID mice (Taconic Farms, New York City, NY) castrated at least one week prior to injection. 500,000 Luciferase expressing LNCaP and LNCaP Enz^R^ cells, or 250,000 CWR-R1 and CWR-R1 Enz^R^ cells, were suspended in 100 μl of PBS (Invitrogen), and injected into the left ventricle of the mice. Metastatic colonization was visualized via Optical Imaging at least once a week post-injection until endpoint was met. Animals were imaged 10 minutes after IP injection of a bolus of Luciferin (Invitrogen) at saturating levels (37.5 mg/kg), once per week following intracardiac injection. Optical Imaging was performed at the integrated Small Animal Imaging Research Resource, Optical Imaging subcore at the University of Chicago on a PerkinElmer IVIS Spectrum with Living Image 4.4 Software.

### Statistical analyses

Data was analyzed using GraphPad Prism software version 5.0 f (GraphPad Software, La Jolla, CA) software, experiments were performed in triplicate to determine mean standard error, and student's *t*-tests performed with normalization to control analyses to obtain *p*-values. For survival analysis, Log-rank (Mantel-Cox) tests were utilized. Growth data was analyzed with *n* = 6 using multiple pair-wise student *t*-tests.

## SUPPLEMENTARY MATERIALS FIGURES AND TABLES



## Abbreviations

ADT: Androgen Deprivation Therapy
AR: Androgen Receptor
CRPC: Castration-Resistant Prostate Cancer
Enz^R^: Enzalutamide Resistant
PC: Prostate Cancer
PSA: Prostate-Specific Antigen
