# Supplementary material for: Acquired resistance to the second-generation androgen receptor antagonist enzalutamide in castration-resistant prostate cancer
Source: Oncotarget. 2016 Mar 29;7(18):26259–74. doi: 10.18632/oncotarget.8456 (PMC5041979; doi:10.18632/oncotarget.8456)
Supplement: Supplementary file 1 [file oncotarget-07-26259-s001.pdf]

Acquired resistance to the second-generation androgen receptor antagonist enzalutamide in castration-resistant prostate cancer

Supplementary Materials

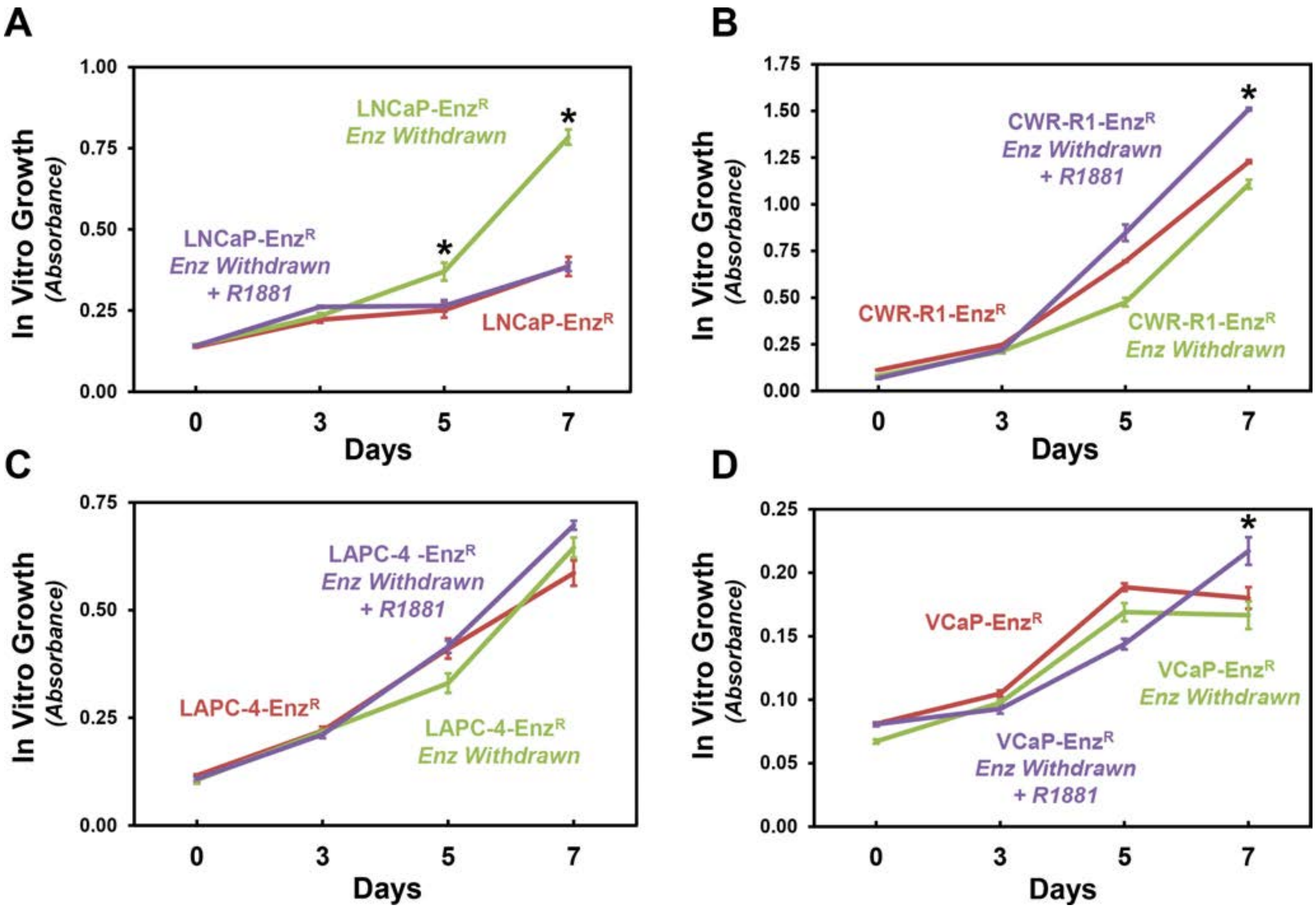

Supplementary Figure S1: Withdrawal of enzalutamide in Enz^R cell lines results in heterogeneous growth responses. Growth rates of LNCaP (**A**), CWR-R1 (**B**), LAPC-4 (**C**), and VCaP (**D**) Enz^R (grown in 10 μM enzalutamide) cells and cells withdrawn from enzalutamide and placed into vehicle control growth media (Enz withdrawn) or cells withdrawn from enzalutamide with 1 nM R1881 added. Cell growth was measured over the course of (7 days) by MTT. Asterisk represent conditions where $p < 0.05$.

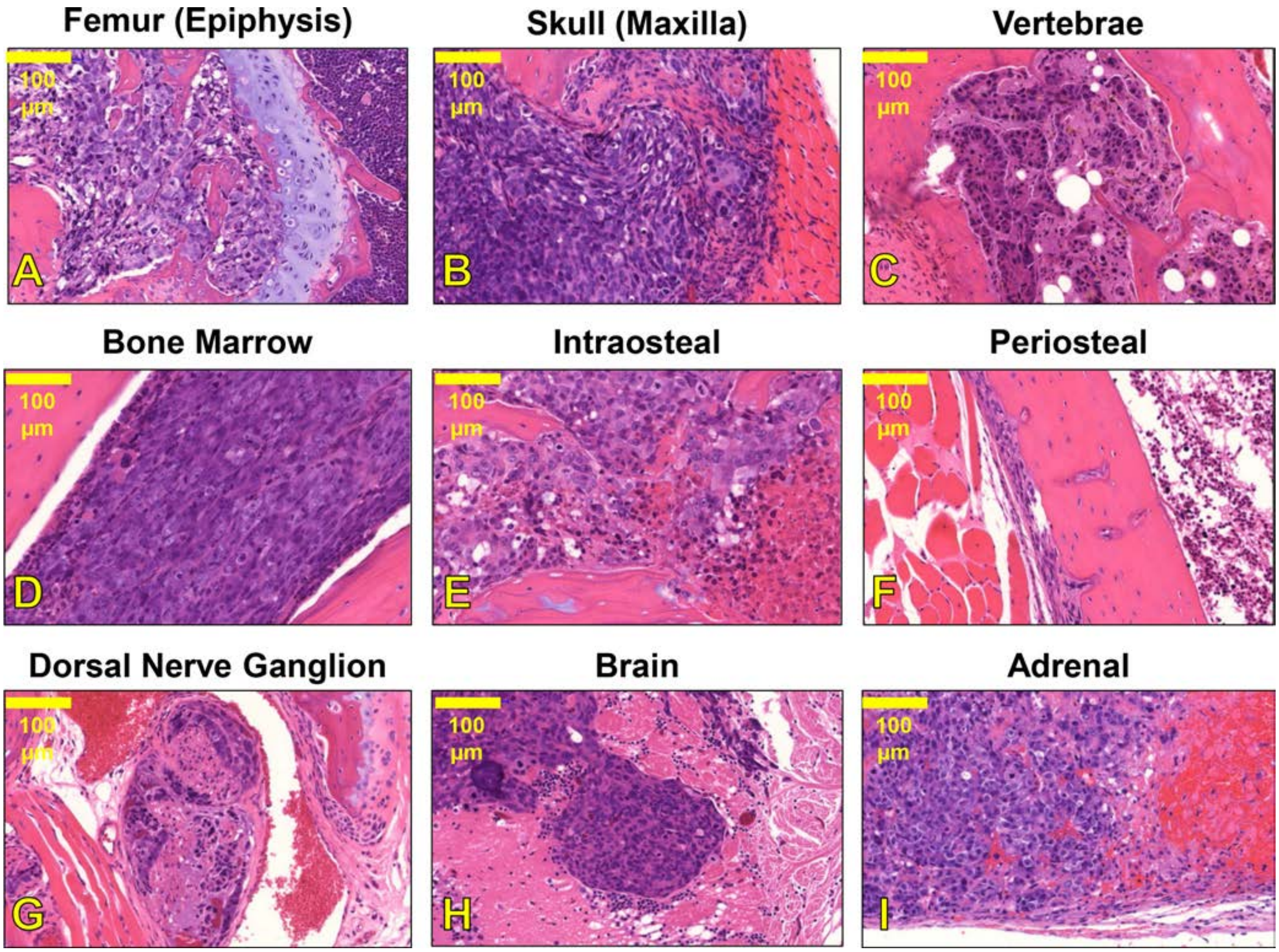

Supplementary Figure S2: Representative images of histological sections of metastases obtained from CWR-R1 Enz^{R} injected mice stained with Hematoxylin and Eosin. Representative images of histological sections stained with Hematoxylin and Eosin from metastases obtained from the CB-17 SCID mice intracardiac injected with 2.5e6 CWR-R1 Enz^{R} cells lentivirally transduced with Promoter-less Luciferase-2 (Promega) after about 4 weeks post injection. Long bone [Femur Epiphysis (**A**)], Skull [Maxilla (**B**)], and Vertebral (**C**) bone metastasis were identified. Bone metastasis formation occurred within the Bone Marrow (**D**), as well as both Intraosteal (**E**) and Periosteal (**F**). Metastatic formation also occurred at the Dorsal Root Nerve Ganglion (**G**), in the Brain (**H**) and Adrenal glands (**I**). All images are at 20× magnification, and were analyzed and identified by a pathologist.

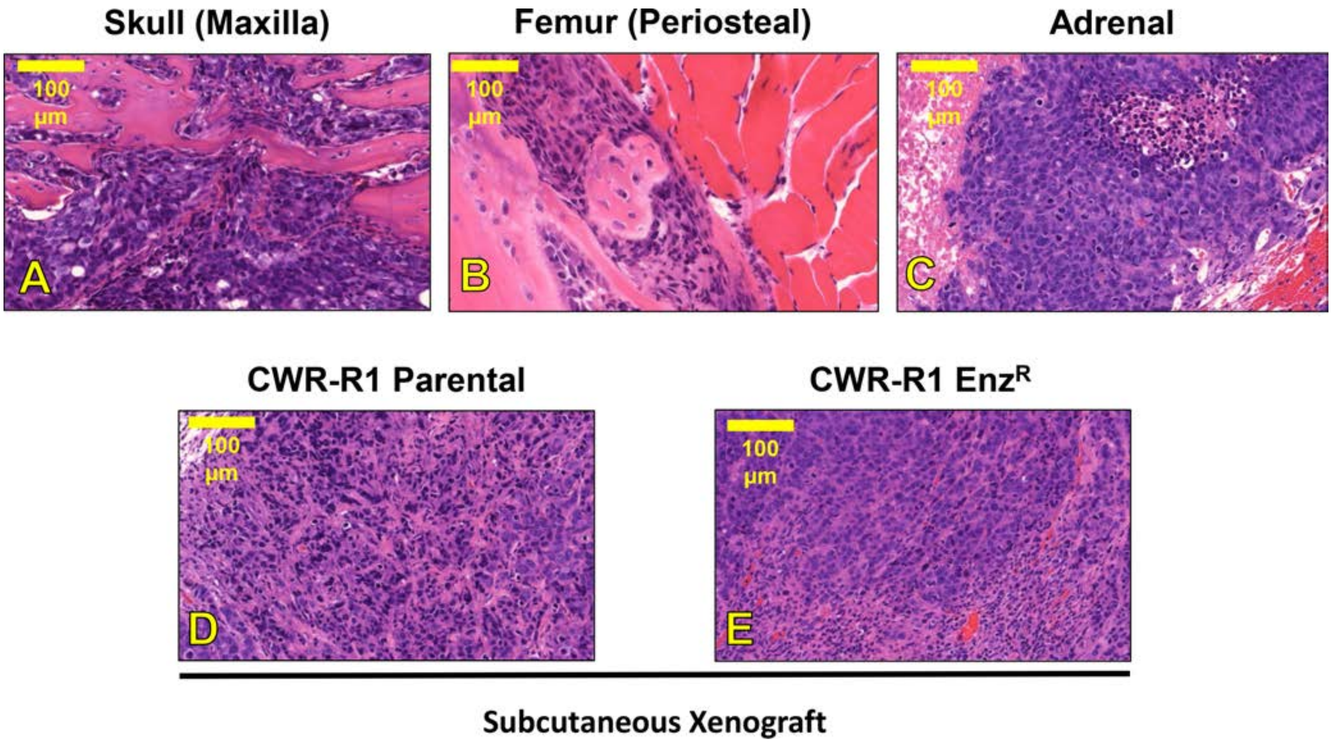

Supplementary Figure S3: Representative images of histological sections of metastases obtained from CWR-R1 injected mice stained with hematoxylin and eosin. Representative images of histological sections stained with Hematoxylin and Eosin from metastases obtained from the CB-17 SCID mice intracardiac injected with 2.5e6 CWR-R1 cells lentivirally transduced with Promoter-less Luciferase-2 (Promega) after about 6 weeks post injection. Similar to CWR-R1 Enz^R cells, Skull [Maxilla (**A**)] and Femoral (**B**) bone metastases were identified, as well as adrenal metastases (**C**). Representative images of subcutaneous tumors both CWR-R1 parental (**D**) and CWR-R1 Enz^R cells (**E**) shows very similar histology between the two cell lines. All images are at 20× magnification, and were analyzed and identified by a pathologist.

Supplementary Table S1: Summary of expression changes between treatment groups

Parental vs. 48 hr Enz	LNCaP	LAPC-4	CWR-R1	VCAP
**Total Gene Changes[a]**	**146**	**334**	**237**	**215**
**Enz Promoted (%)** *(Expression Increases in Response to Enz)*	91 (62%)	132 (39%)	122 (51%)	127 (59%)
**Enz Inhibited (%)** *(Expression Decreases in Response to Enz)*	55 (38%)	212 (61%)	115 (49%)	88 (41%)

Parental vs. EnzR	LNCaP	LAPC-4	CWR-R1	VCAP
**Total Gene Changes[a]**	**1069**	**985**	**404**	**649**
**Expression in EnzR Increased (%)**	645 (60%)	527 (54%)	199 (49%)	362 (56%)
**Expression in EnzR Decreased (%)**	424 (40%)	458 (46%)	205 (51%)	287 (44%)

48 hr Enz vs. Enz^R	LNCaP	LAPC-4	CWR-R1	VCAP
**Total Gene Changes[a]**	**391**	**26**	**221**	**238**
**Overlap with Parental vs. 48 hr Enz Genes** *(AR-Pathway Restored genes)* *(% of total)*	146 (37%)	23 (88%)	116 (52%)	143 (60%)
**Enz Promoted, Further Repressed** *(Expression increases further in EnzR)*	64	6	70	42
**Enz Inhibited, Further Promoted** *(Expression decreases further in EnzR)*	31	2	12	30
**Enz Promoted, Expression Reversed** *(Expression Reversed in EnzR)*	27	7	13	41
**Enz Inhibited, Expression Reversed** *(Expression Reversed in EnzR)*	24	8	21	30
**No Overlap with Parental vs. 48 hr Enz Genes** *(Novel non-AR-associated genes)* *(% of total)*	245 (63%)	3 (12%)	105 (48%)	95 (40%)
**Novel Increased Genes in EnzR**	103	1	53	43
**Novel Decreased Genes Decreased in EnzR**	142	2	52	52

a: Changes represent differentially expressed genes using the Significance Analysis of Microarrays (SAM), which performs appropriate tests for multiplicity. Genes that met a 5% false discovery rate (FDR) were retained.

Supplementary Table S2: Gene expression reversal of AR-Associated genes

			LNCaP		LAPC-4		CWR-R1		VCaP	
**Symbol**	**Gene Name**	**Entrez ID**	**Parental vs. 48 hr Enz**	**48 hr vs. EnzR**	**Parental vs. 48 hr Enz**	**48 hr vs. EnzR**	**Parental vs. 48 hr Enz**	**48 hr vs. EnzR**	**Parental vs. 48 hr Enz**	**48 hr vs. EnzR**
**Column1**	**Column 2**	**Column 3**	**Column 4**	**Column 5**	**Column 6**	**Column 7**	**Column 8**	**Column 9**	**Column 10**	**Column 11**
ABCA1	ATP-binding cassette, sub-family A (ABC1), member 1	*19*			1.31	0.81				
ABCC4	ATP-binding cassette, sub-family C (CFTR/MRP), member 4	*10257*					1.04	0.90		
ACLY	aminoacylase 1	*95*	0.25	1.37						
ACSM3	acyl-CoA synthetase medium-chain family member 3	*6296*	1.32	0.39						
ACTA2	actin, alpha 2, smooth muscle, aorta	*59*					0.87	1.07		
ADD3	adducin 3 (gamma)	*120*	0.76	2.19						
ADM	adrenomedullin	*133*					1.22	0.63		
AGA	aspartylglucosaminidase	*175*	1.78	0.84						
AGR2	anterior gradient homolog 2 (Xenopus laevis)	*10551*							1.75	0.43
AKR1C3	aldo-keto reductase family 1, member C3 (3-alpha hydroxysteroid dehydrogenase, type II)	*8644*							1.14	0.41
ALB	albumin	*213*					0.88	1.81		
ALDH1A1	aldehyde dehydrogenase 1 family, member A1	*216*							0.65	2.17
ALDH1A3	aldehyde dehydrogenase 1 family, member A3	*220*					0.78	1.11	1.23	0.54
ANG	angiogenin, ribonuclease, RNase A family, 5	*283*	1.23	0.79						
ANXA1	annexin A1	*301*			1.28	0.75			1.55	0.45
APOD	apolipoprotein D	*347*	0.30	1.25						
ATP1B1	ATPase, Na+/K+ transporting, beta 1 polypeptide	*481*							1.75	0.72
BAMBI	BMP and activin membrane-bound inhibitor homolog (Xenopus laevis)	*25805*	1.39	0.33					2.43	0.45
BCL2L12	BCL2-like 12 (proline rich)	*83596*							0.92	1.17
BRP44	brain protein 44	*25874*	2.23	0.78					0.75	1.38
BTG3	BTG family, member 3	*10950*							0.87	1.77
C12orf24	chromosome 12 open reading frame 24	*29902*	1.28	0.65						
C19orf48	chromosome 19 open reading frame 48	*84798*							0.75	1.22
C1orf116	chromosome 1 open reading frame 116	*79098*	1.31	0.71	0.11	1.31				
C3orf14	chromosome 3 open reading frame 14	*57415*			0.70	1.18				
C3orf58	chromosome 3 open reading frame 58	*205428*							0.69	1.20
C7orf63	chromosome 7 open reading frame 63	*79846*					0.90	1.09		
C9orf152	chromosome 9 open reading frame 152	*401546*							1.21	0.64
CAMK2N1	calcium/calmodulin-dependent protein kinase II inhibitor 1	*55450*					1.18	0.89		
CCNO	cyclin O	*10309*					2.17	0.58		
CD24	CD24 molecule	*100133941*							1.15	0.63
CD9	CD9 molecule	*928*							1.23	0.75
CDH2	cadherin 2, type 1, N-cadherin (neuronal)	*1000*							1.14	0.81

CDK19	cyclin-dependent kinase 19	*23097*					1.17	0.86		
CENPN	centromere protein N	*55839*	1.35	0.87						
CHST15	carbohydrate (N-acetylgalactosamine 4-sulfate 6-O) sulfotransferase 15	*51363*							1.21	0.85
CKS1B	CDC28 protein kinase regulatory subunit 1B	*1163*	1.61	0.57						
COL4A5	collagen, type IV, alpha 5	*1287*					0.85	1.22	0.89	1.40
COLEC12	collectin sub-family member 12	*81035*	0.79	3.29						
CRIP2	cysteine-rich protein 2	*1397*					1.15	0.73		
CXCR7	chemokine (C-X-C motif) receptor 7	*57007*	0.30	1.18			1.30	0.81		
CXXC5	CXXC finger protein 5	*51523*							1.20	0.81
CYB5A	cytochrome b5 type A (microsomal)	*1528*							0.73	1.77
CYP4V2	cytochrome P450, family 4, subfamily V, polypeptide 2	*285440*							1.18	0.85
CYP4X1	cytochrome P450, family 4, subfamily X, polypeptide 1	*260293*							1.60	0.84
DBT	dihydrolipoamide branched chain transacylase E2	*1629*							0.90	1.21
DDIT4L	DNA-damage-inducible transcript 4	*54541*							0.88	1.15
DLX1	distal-less homeobox 1	*1745*					0.89	1.12		
EEF1A2	eukaryotic translation elongation factor 1 alpha 2	*1917*							1.19	0.81
EHF	ets homologous factor	*26298*	1.77	0.81						
ELOVL4	elongation of very long chain fatty acids (FEN1/ Elo2, SUR4/Elo3, yeast)-like 4	*6785*					1.17	0.78		
ENDOD1	endonuclease domain containing 1	*23052*					0.68	1.09		
EXO1	exonuclease 1	*9156*	2.04	0.76						
FAM111A	family with sequence similarity 111, member A	*63901*	0.64	1.13						
FAM174B	family with sequence similarity 174, member B	*400451*	0.63	1.42						
FKBP5	FK506 binding protein 5	*2289*	1.29	0.79					0.54	1.90
FZD4	frizzled homolog 4 (Drosophila)	*8322*							1.05	0.77
GCNT1	glucosaminyl (N-acetyl) transferase 1, core 2	*2650*	5.51	0.51						
GUCY1A3	guanylate cyclase 1, soluble, alpha 3	*2982*					0.72	1.17	0.82	1.32
H2AFJ	H2A histone family, member J	*55766*					0.80	1.39		
HBQ1	hemoglobin, theta 1	*3049*	0.78	1.18			1.06	0.94	0.83	1.23
HIBADH	3-hydroxyisobutyrate dehydrogenase	*11112*	1.46	0.63						
HIST1H2BK	histone cluster 1, H2bk	*85236*							1.15	0.65
HIST2H2BE	histone cluster 2, H2be	*8349*							1.12	0.80
HLA-A	major histocompatibility complex, class I, A	*3105*							1.12	0.67
HOMER2	homer homolog 2 (Drosophila)	*9455*			0.36	1.19				
ID1	inhibitor of DNA binding 1, dominant negative helix-loop-helix protein	*3397*	4.44	0.76						
ID2	inhibitor of DNA binding 2, dominant negative helix-loop-helix protein	*3398*							1.54	0.66

ID3	inhibitor of DNA binding 3, dominant negative helix-loop-helix protein	*3399*	1.23	0.67						
IER3	immediate early response 3	*8870*							1.13	0.61
IFITM3	interferon induced transmembrane protein 3 (1-8U)	*10410*							1.22	0.80
IGFBP3	insulin-like growth factor binding protein 3	*3486*							1.58	0.12
IGFBP5	insulin-like growth factor binding protein 5	*3488*			3.93	0.76			1.19	0.64
KCNK1	potassium channel, subfamily K, member 1	*3775*					1.10	0.92		
KLK2	kallikrein-related peptidase 2	*3817*			0.18	1.17				
KLK4	kallikrein-related peptidase 4	*9622*	1.28	0.42	0.18	1.57			0.63	3.58
LAD1	ladinin 1	*3898*			0.69	1.23				
LCP1	lymphocyte cytosolic protein 1 (L-plastin)	*3936*							0.88	1.28
LIMCH1	LIM and calponin homology domains 1	*22998*							1.60	0.78
LITAF	lipopolysaccharide-induced TNF factor	*9516*			1.17	0.86			1.09	0.53
LMO4	LIM domain only 4	*8543*			2.29	0.77			1.27	0.71
LRRC31	leucine rich repeat containing 31	*79782*			1.33	0.70				
LRRN1	leucine rich repeat neuronal 1	*57633*	0.75	1.18						
LY6E	lymphocyte antigen 6 complex, locus E	*4061*	1.27	0.80						
MAOA	monoamine oxidase A	*4128*							0.63	7.36
MCCC2	methylcrotonoyl-CoA carboxylase 2 (beta)	*64087*	0.40	2.96			0.43	2.13		
MDK	midkine (neurite growth-promoting factor 2)	*4192*					0.88	1.40		
MKNK2	MAP kinase interacting serine/threonine kinase 2	*2872*	0.75	1.32						
MME	membrane metallo-endopeptidase	*4311*	0.76	1.41					0.86	1.31
MNS1	meiosis-specific nuclear structural 1	*55329*	1.25	0.69						
MSX1	msh homeobox 1	*4487*							0.76	2.70
MT1G	metallothionein 1G	*4495*							0.79	2.21
NBL1	neuroblastoma, suppression of tumorigenicity 1	*4681*							1.12	0.80
NFKBIA	nuclear factor of kappa light polypeptide gene enhancer in B-cells inhibitor, alpha	*4792*							1.27	0.79
NLGN4X	neuroligin 4, X-linked	*57502*			1.83	0.86				
PAK1IP1	PAK1 interacting protein 1	*55003*	1.41	0.72						
PALM	paralemmin	*5064*							1.19	0.83
PBK	PDZ binding kinase	*55872*							0.85	1.23
PDE9A	phosphodiesterase 9A	*5152*							0.79	1.41
PFKFB3	6-phosphofructo-2-kinase/fructose-2,6-biphosphatase 3	*5209*	0.69	1.84						
PFKFB4	6-phosphofructo-2-kinase/fructose-2,6-biphosphatase 4	*5210*	0.70	3.43						
PFKP	phosphofructokinase, platelet	*5214*	0.74	2.40						
PLA2G3	phospholipase A2, group III	*50487*							1.17	0.77
PLA2G4C	phospholipase A2, group IVC (cytosolic, calcium-independent)	*8605*					0.77	1.18		

PMEPA1	prostate transmembrane protein, androgen induced 1	*56937*							0.80	3.16
PNPLA7	patatin-like phospholipase domain containing 7	*375775*							1.30	0.81
PPAP2A	phosphatidic acid phosphatase type 2A	*8611*	1.70	0.26						
PPIC	peptidylprolyl isomerase C (cyclophilin C)	*5480*			2.17	0.79				
PPP1R1B	protein phosphatase 1, regulatory (inhibitor) subunit 1B	*84152*							1.18	0.60
PRKACB	protein kinase, cAMP-dependent, catalytic, beta	*5567*					0.81	1.11		
PRUNE2	prune homolog 2 (Drosophila)	*158471*					0.79	1.86		
PTTG3P	pituitary tumor-transforming 3 (pseudogene)	*26255*	1.33	0.80						
RAP1GAP	RAP1 GTPase activating protein	*5909*							1.18	0.57
RHOBTB3	Rho-related BTB domain containing 3	*22836*							0.76	1.33
RPL13A	ribosomal protein L13a	*23521*							1.11	0.86
RPL29	ribosomal protein L29	*6159*	1.46	0.78						
SAT1	spermidine/spermine N1-acetyltransferase 1	*6303*							1.24	0.81
SBK1	SH3-binding domain kinase 1	*388228*					0.91	1.28		
SELENBP1	selenium binding protein 1	*8991*	0.29	1.75						
SERPINI1	serpin peptidase inhibitor, clade I (neuroserpin), member 1	*5274*	0.78	1.49						
SHROOM3	shroom family member 3	*57619*							0.88	1.20
SLC16A3	solute carrier family 16, member 3 (monocarboxylic acid transporter 4)	*9123*					0.89	1.06		
SLC27A3	solute carrier family 27 (fatty acid transporter), member 3	*11000*							1.19	0.84
SLC45A3	solute carrier family 45, member 3	*85414*	1.32	0.55						
SLC6A10P	solute carrier family 6 (neurotransmitter transporter, creatine), member 10 (pseudogene)	*386757*	0.82	1.25						
SOCS2	suppressor of cytokine signaling 2	*8835*					1.58	0.70	0.50	1.36
SPRYD5	SPRY domain containing 5	*84767*	0.69	1.18						
STEAP1	six transmembrane epithelial antigen of the prostate 1	*26872*							0.56	1.22
STEAP2	six transmembrane epithelial antigen of the prostate 2	*261729*							0.84	1.43
STK39	serine threonine kinase 39	*27347*							0.52	1.28
TAGLN2	transgelin 2	*8407*	0.78	1.33						
TFAP2A	transcription factor AP-2 alpha (activating enhancer binding protein 2 alpha)	*7020*					0.87	1.08		
TFF3	trefoil factor 3 (intestinal)	*7033*					0.89	1.29		
TFPI	tissue factor pathway inhibitor (lipoprotein-associated coagulation inhibitor)	*7035*	0.59	2.38						
THNSL2	threonine synthase-like 2 (S. cerevisiae)	*55258*							1.17	0.81

TMEFF2	transmembrane protein with EGF-like and two follistatin-like domains 2	*23671*	0.55	11.49	0.17	1.14	0.37	2.19	0.57	1.63
TMPRSS2	transmembrane protease, serine 2	*7113*	0.20	1.28						
TMSB15A	thymosin beta 15a	*11013*	0.73	1.53			0.84	1.08		
TMSB15B	thymosin beta 15B	*286527*	0.77	1.28			0.91	1.10		
TNFRSF19	tumor necrosis factor receptor superfamily, member 19	*55504*							1.34	0.47
TP53INP1	tumor protein p53 inducible nuclear protein 1	*94241*	1.66	0.85						
TPD52	tumor protein D52	*7163*	0.78	1.22						
UGT2B11	UDP glucuronosyltransferase 2 family, polypeptide B11	*10720*	0.69	1.19						
UGT2B15	UDP glucuronosyltransferase 2 family, polypeptide B15	*7366*					1.13	0.64		
UGT2B17	UDP glucuronosyltransferase 2 family, polypeptide B17	*7367*					1.18	0.69		
ZIC2	Zic family member 2 (odd-paired homolog, Drosophila)	*7546*							1.14	0.83
ZNF385B	zinc finger protein 385B	*151126*							0.52	1.79
ZNF395	zinc finger protein 395	*55893*	0.76	1.23						
ZSCAN18	zinc finger and SCAN domain containing 18	*65982*							1.18	0.81

Parental vs. 48 hr Enz: fold-change mRNA expression in response to acute Enzalutamide
48 hr vs. EnzR: fold-change mRNA expression in response to acquistion of Enzalutamide Resistance

Supplementary Table S3: Gene expression of Non-AR associated genes in Enz^R cells

Symbol	Gene Name	Entrez ID	LNCaP	LAPC-4	CWR-R1	VCaP
**Column1**	**Column2**	**Column3**	**Column4**	**Column5**	**Column6**	**Column7**
ACADVL	acyl-CoA dehydrogenase, very long chain	*37*	**1.16**			
ACOT13	acyl-CoA thioesterase 13	*55856*	**0.720**			
ACY1	aminoacylase 1	*95*				**1.31**
ADAM15	ADAM metallopeptidase domain 15	*8751*	**0.760**			
ADAMTS1	ADAM metallopeptidase with thrombospondin type 1 motif, 1	*9510*	**7.54**			**1.05**
ADD3	adducin 3 (gamma)	*120*			**1.71**	
AGR2	anterior gradient homolog 2 (Xenopus laevis)	*10551*	**1.11**			**0.54**
AK3	adenylate kinase 3	*50808*	**1.73**			
AKR1C3	aldo-keto reductase family 1, member C3 (3-alpha hydroxysteroid dehydrogenase, type II)	*8644*	**0.600**			
ALCAM	activated leukocyte cell adhesion molecule	*214*	**1.56**		**0.770**	
ALDH3B2	aldehyde dehydrogenase 3 family, member B2	*222*				**1.18**
ALDOA	aldolase A, fructose-bisphosphate	*226*	**1.77**			
ALDOC	aldolase C, fructose-bisphosphate	*230*	**1.72**		**0.760**	
AMACR	alpha-methylacyl-CoA racemase	*23600*	**0.52**			
ANG	angiogenin, ribonuclease, RNase A family, 5	*283*				**1.49**
ANGPTL4	angiopoietin-like 4	*51129*				**0.890**
ANKRD37	ankyrin repeat domain 37	*353322*				**0.920**
APP	amyloid beta (A4) precursor protein	*351*	**1.94**		**0.53**	
ARL2	ADP-ribosylation factor-like 2	*402*	**1.31**			**1.23**
ASAP3	ArfGAP with SH3 domain, ankyrin repeat and PH domain 3	*55616*	**1.29**			
ASNS	asparagine synthetase (glutamine-hydrolyzing)	*440*	**0.830**			
ASPM	asp (abnormal spindle) homolog, microcephaly associated (Drosophila)	*259266*			**0.790**	
ATP2A2	ATPase, Ca++ transporting, cardiac muscle, slow twitch 2	*488*				**0.820**
ATP6V0E2	ATPase, H+ transporting V0 subunit e2	*155066*			**1.23**	**0.830**
BARD1	BRCA1 associated RING domain 1	*580*			**1.34**	
BASP1	brain abundant, membrane attached signal protein 1	*10409*	**0.11**			
BCL6	B-cell CLL/lymphoma 6	*604*	**0.840**			**0.830**

BCYRN1	brain cytoplasmic RNA 1 (non-protein coding)	*618*	**1.41**		**1.55**	
BEX1	brain expressed, X-linked 1	*55859*			**0.430**	
BEX2	brain expressed X-linked 2	*84707*			**0.810**	
BIRC5	baculoviral IAP repeat containing 5	*332*	**0.810**	1.22		
BLVRA	biliverdin reductase A	*644*	**1.46**			
BNIP3	BCL2/adenovirus E1B 19kDa interacting protein 3	*664*	**2.68**			
BNIP3L	BCL2/adenovirus E1B 19kDa interacting protein 3-like	*665*	**1.46**			
BSCL2	Berardinelli-Seip congenital lipodystrophy 2 (seipin)	*26580*	**1.27**		**1.42**	
C10orf58	chromosome 10 open reading frame 58	*84293*	**1.74**			
C11orf1	chromosome 11 open reading frame 1	*64776*	**0.840**			
C14orf159	chromosome 14 open reading frame 159	*80017*	**0.810**			
C20orf108	chromosome 20 open reading frame 108	*116151*	**0.770**			
C7orf63	chromosome 7 open reading frame 63	*79846*	**1.25**			
C9orf152	chromosome 9 open reading frame 152	*401546*	**1.22**			**1.3**
CA12	carbonic anhydrase XII	*771*	**0.650**			**0.750**
CALD1	caldesmon 1	*800*	**1.4**			
CALU	calumenin	*813*				**0.830**
CAMK2N1	calcium/calmodulin-dependent protein kinase II inhibitor 1	*55450*			**1.58**	
CAP2	CAP, adenylate cyclase-associated protein, 2 (yeast)	*10486*	**0.45**		**1.53**	
CBX2	chromobox homolog 2	*84733*			**0.690**	
CCDC109B	coiled-coil domain containing 109B	*55013*	**1.16**			
CCDC53	coiled-coil domain containing 53	*51019*	**1.28**			
CCND1	cyclin D1	*595*	**2.02**			
CCNF	cyclin F	*899*	**1.14**		**1.15**	
CCNO	cyclin O	*10309*	**1.15**			
CD163L1	CD163 molecule-like 1	*283316*	**1.28**			**1.06**
CD24	CD24 molecule	*100133941*	**0.3**			
CD9	CD9 molecule	*928*	**1.27**			
CDC16	cell division cycle 16 homolog (S. cerevisiae)	*8881*	**1.17**			
CDH3	cadherin 3, type 1, P-cadherin (placental)	*1001*			**1.15**	
CDK19	cyclin-dependent kinase 19	*23097*	**0.740**			

CDKN1A	cyclin-dependent kinase inhibitor 1A (p21, Cip1)	*1026*				**0.810**
CENPN	centromere protein N	*55839*			**1.22**	
CENPV	centromere protein V	*201161*	**1.51**			
CGNL1	cingulin-like 1	*84952*	**2.07**			
CHPT1	choline phosphotransferase 1	*56994*	**0.740**		**2.27**	**0.830**
CHST15	carbohydrate (N-acetylgalactosamine 4-sulfate 6-O) sulfotransferase 15	*51363*	**0.810**			
CIB1	calcium and integrin binding 1 (calmyrin)	*10519*			**1.29**	**0.900**
CKB	creatine kinase, brain	*1152*	**8.76**		**1.23**	
CLEC11A	C-type lectin domain family 11, member A	*6320*	**0.8**			
COBLL1	COBL-like 1	*22837*	**2.19**		**1.21**	
COL4A5	collagen, type IV, alpha 5	*1287*	**0.45**			
CRIP2	cysteine-rich protein 2	*1397*	**1.64**			
CSRP1	cysteine and glycine-rich protein 1	*1465*	**1.19**			
CTAG2	cancer/testis antigen 2	*30848*			**0.850**	
CTBS	chitobiase, di-N-acetyl-	*1486*	**1.49**			
CXADR	coxsackie virus and adenovirus receptor	*1525*	**1.36**		**1.28**	**1.2**
CYB5A	cytochrome b5 type A (microsomal)	*1528*	**0.740**			
CYP2J2	cytochrome P450, family 2, subfamily J, polypeptide 2	*1573*	**0.430**			**0.880**
CYP4V2	cytochrome P450, family 4, subfamily V, polypeptide 2	*285440*	**0.710**			
DBT	dihydrolipoamide branched chain transacylase E2	*1629*	**1.5**			
DDIT4	DNA-damage-inducible transcript 4	*54541*				**0.620**
DENND1A	DENN/MADD domain containing 1A	*57706*	**1.77**			
DHRS7	dehydrogenase/reductase (SDR family) member 7	*51635*			**1.21**	
DLL1	delta-like 1 (Drosophila)	*28514*			**0.760**	
DLL3	delta-like 3 (Drosophila)	*10683*			**0.750**	
DOPEY2	dopey family member 2	*9980*				**1.53**
DPM3	dolichyl-phosphate mannosyltransferase polypeptide 3	*54344*	**0.64**			
DSC2	desmocollin 2	*1824*				**1.19**
DYNC1LI2	dynein, cytoplasmic 1, light intermediate chain 2	*1783*	**1.22**			
E2F2	E2F transcription factor 2	*1870*	**0.870**			
ECHDC2	enoyl CoA hydratase domain containing 2	*55268*	**1.27**			**1.13**

EEF1A2	eukaryotic translation elongation factor 1 alpha 2	*1917*	**1.21**			**1.22**
EIF1AY	eukaryotic translation initiation factor 1A, Y-linked	*9086*	**0.8**			
EIF2S3	eukaryotic translation initiation factor 2, subunit 3 gamma, 52kDa	*1968*	**0.840**		**0.830**	
ENO2	enolase 2 (gamma, neuronal)	*2026*	**2.12**		**1.3**	**1.13**
FAM174B	family with sequence similarity 174, member B	*400451*			**0.760**	
FAM46A	family with sequence similarity 46, member A	*55603*	**0.840**			
FAR2	fatty acyl CoA reductase 2	*55711*			**1.23**	
FGGY	FGGY carbohydrate kinase domain containing	*55277*	**1.32**			**1.14**
FKBP11	FK506 binding protein 11, 19 kDa	*51303*			**1.17**	**0.770**
FLRT3	fibronectin leucine rich transmembrane protein 3	*23767*			**1.11**	
FZD2	frizzled homolog 2 (Drosophila)	*2535*	**1.15**			
FZD4	frizzled homolog 4 (Drosophila)	*8322*	**0.750**			**1.17**
GABRB3	gamma-aminobutyric acid (GABA) A receptor, beta 3	*2562*	**0.730**			
GAGE12B	G antigen 12B	*729428*	**0.490**			
GAGE12C	G antigen 12C	*729422*	**0.58**			
GAGE12E	G antigen 12E	*729431*	**0.58**			
GAGE12F	G antigen 12F	*100008586*	**0.600**			
GAGE12G	G antigen 12G	*645073*	**0.680**			
GAGE12H	G antigen 12H	*729442*	**0.58**			
GAGE12I	G antigen 12I	*26748*	**0.64**			
GAGE12J	G antigen 12J	*729396*	**0.650**			
GAGE2A	G antigen 2A	*729447*	**0.550**			
GAGE2B	G antigen 2B	*645037*	**0.650**			
GAGE2E	G antigen 2E	*26749*	**0.510**			
GAGE4	G antigen 4	*2576*	**0.680**			
GAGE5	G antigen 5	*2577*	**0.630**			
GAGE6	G antigen 6	*2578*	**0.600**			
GALK1	galactokinase 1	*2584*	**3.68**		**1.56**	
GALT	galactose-1-phosphate uridylyltransferase	*2592*				**1.21**
GAMT	guanidinoacetate N-methyltransferase	*2593*			**1.34**	
GLDC	glycine dehydrogenase (decarboxylating)	*2731*	**1.32**			
GNAS	GNAS complex locus	*2778*	**1.34**			
GNG10	guanine nucleotide binding protein (G protein), gamma 10	*2790*	**1.44**			
GNG5	guanine nucleotide binding protein (G protein), gamma 5	*2787*	**0.710**			

GPD1L	glycerol-3-phosphate dehydrogenase 1-like	*23171*	**0.730**			
GPX1	glutathione peroxidase 1	*2876*			**1.56**	**1.2**
GSR	glutathione reductase	*2936*	**0.710**			
GSTO2	glutathione S-transferase omega 2	*119391*				**1.19**
GTF2E2	general transcription factor IIE, polypeptide 2, beta 34kDa	*2961*	**1.36**			
GUCY1A3	guanylate cyclase 1, soluble, alpha 3	*2982*	**2.77**			
H2AFJ	H2A histone family, member J	*55766*	**10.25**			
H3F3B	H3 histone, family 3B (H3.3B)	*3021*	**1.36**			
HENMT1	HEN1 methyltransferase homolog 1 (Arabidopsis)	*113802*	**3.39**		**0.8**	
HGD	homogentisate 1,2-dioxygenase	*3081*	**0.840**			
HIST1H1C	histone cluster 1, H1c	*3006*				**1.31**
HIST1H2BK	histone cluster 1, H2bk	*85236*	**1.32**			**1.17**
HLA-A	major histocompatibility complex, class I, A	*3105*	**0.590**			**0.790**
HLA-DMB	major histocompatibility complex, class II, DM beta	*3109*	**2.03**			
HOXA11-AS1	HOXA11 antisense RNA 1 (non-protein coding)	*221883*	**0.52**			**1.27**
HOXA5	HOXA11 antisense RNA 1 (non-protein coding)	*221883*	**1.71**		**1.24**	
HOXA9	homeobox A9	*3205*			**1.29**	
HOXC6	homeobox C6	*3223*	**1.84**		**2.15**	
HSD17B12	hydroxysteroid (17-beta) dehydrogenase 12	*51144*	**1.28**		**0.820**	
HSPA5	heat shock 70kDa protein 5 (glucose-regulated protein, 78kDa)	*3309*	**1.34**			**0.8**
HSPB1	heat shock 27kDa protein 1	*3315*			**2.53**	
ID2	inhibitor of DNA binding 2, dominant negative helix-loop-helix protein	*3398*	**0.690**			
IFITM2	interferon induced transmembrane protein 2 (1-8D)	*10581*	**1.74**		**0.700**	**0.900**
IGF2BP3	insulin-like growth factor 2 mRNA binding protein 3	*10643*	**1.12**			
IGF2R	insulin-like growth factor 2 receptor	*3482*	**2.17**			
IGFBP3	insulin-like growth factor binding protein 3	*3486*	**0.14**			
IGFBP5	insulin-like growth factor binding protein 5	*3488*				**0.930**
IGSF21	immunoglobin superfamily, member 21	*84966*	**0.8**			
ILVBL	ilvB (bacterial acetolactate synthase)-like	*10994*				**0.870**

INF2	inverted formin, FH2 and WH2 domain containing	*64423*	**1.3**			
IRS2	insulin receptor substrate 2	*8660*			**0.730**	
ISG20	interferon stimulated exonuclease gene 20kDa	*3669*	**1.19**		**0.78**	**0.900**
ITGB5	integrin, beta 5	*3693*			**0.740**	
KCNK1	potassium channel, subfamily K, member 1	*3775*	**1.22**			
KIAA1324	KIAA1324	*57535*	**0.34**			
KIF1A	kinesin family member 1A	*547*	**1.9**			
KIF1B	kinesin family member 1B	*23095*	**1.34**			
KIF5C	kinesin family member 5C	*3800*	**1.26**			
KLHL35	kelch-like 35 (Drosophila)	*283212*	**0.720**		**0.690**	
LACTB2	lactamase, beta 2	*51110*	**1.35**		**1.41**	
LAD1	ladinin 1	*3898*	**0.57**		**0.690**	**0.870**
LDOC1	leucine zipper, down-regulated in cancer 1	*23641*	**0.660**			
LEF1	lymphoid enhancer-binding factor 1	*51176*	**0.490**			**1.51**
LEPREL1	leprecan-like 1	*55214*				**1.1**
LIMCH1	LIM and calponin homology domains 1	*22998*	**0.660**			
LMO4	LIM domain only 4	*8543*	**0.730**			**1.14**
LOC497256	hypothetical LOC497256	*497256*	**2.7**			
LOC653506	similar to meteorin, glial cell differentiation regulator-like	*653506*	**1.31**		**0.670**	
LOC729887	hypothetical protein LOC729887	*729887*	**0.860**			**1.04**
LPAR2	lysophosphatidic acid receptor 2	*9170*	**0.820**		**0.850**	
LPL	lipoprotein lipase	*4023*				**0.430**
LYRM5	LYR motif containing 5	*144363*	**1.24**		**1.31**	
LYSMD2	LysM, putative peptidoglycan-binding, domain containing 2	*256586*	**0.830**			
MAGEA1	melanoma antigen family A, 1 (directs expression of antigen MZ2-E)	*4100*	**1.88**			
MAGED1	melanoma antigen family D, 1	*9500*	**1.24**			
MAOA	monoamine oxidase A	*4128*	**5.02**			
MAP1LC3A	microtubule-associated protein 1 light chain 3 alpha	*84557*	**1.3**			
MBP	myelin basic protein	*4155*	**2.69**			
MDK	midkine (neurite growth-promoting factor 2)	*4192*	**1.85**			
ME1	malic enzyme 1, NADP(+)-dependent, cytosolic	*4199*	**0.670**			**0.840**
MFGE8	milk fat globule-EGF factor 8 protein	*4240*	**1.54**		**0.790**	
MGST1	microsomal glutathione S-transferase 1	*4257*	**0.730**		**2.63**	

MIDN	midnolin	*90007*	**1.36**			
MIPEP	mitochondrial intermediate peptidase	*4285*	**1.64**		**0.670**	
MLPH	melanophilin	*79083*				**1.27**
MT1E	metallothionein 1E	*4493*	**1.47**		**1.89**	
MT1G	metallothionein 1G	*4495*	**8.43**			
MT1X	metallothionein 1X	*4501*	**1.51**		**1.48**	
MT2A	metallothionein 2A	*4502*	**1.22**	0.690	**1.67**	**0.58**
NBL1	neuroblastoma, suppression of tumorigenicity 1	*4681*				**1.39**
NCAM2	neural cell adhesion molecule 2	*4685*	**1.65**			
NDRG1	N-myc downstream regulated 1	*10397*				**0.670**
NET1	neuroepithelial cell transforming 1	*10276*	**1.21**		**0.630**	
NFKBIA	nuclear factor of kappa light polypeptide gene enhancer in B-cells inhibitor, alpha	*4792*	**0.670**			
NINJ1	ninjurin 1	*4814*	**1.16**			**0.900**
NKX3-1	NK3 homeobox 1	*4824*			**0.700**	
NLGN4X	neuroligin 4, X-linked	*57502*	**2.92**			
NME4	NME/NM23 nucleoside diphosphate kinase 4	*4833*			**0.850**	
NQO1	NAD(P)H dehydrogenase, quinone 1	*1728*	**0.460**			
OPLAH	5-oxoprolinase (ATP-hydrolysing)	*26873*	**0.820**			
P4HA1	prolyl 4-hydroxylase, alpha polypeptide I	*5033*	**1.86**			
PACSIN1	protein kinase C and casein kinase substrate in neurons 1	*29993*	**1.38**			
PALM	paralemmin	*5064*	**1.27**			
PAM	peptidylglycine alpha-amidating monooxygenase	*5066*	**0.490**		**0.770**	**1.14**
PARP10	poly (ADP-ribose) polymerase family, member 10	*84875*	**0.700**			
PARP3	poly (ADP-ribose) polymerase family, member 3	*10039*	**0.810**			
PBDC1	polysaccharide biosynthesis domain containing 1	*51260*			**0.820**	
PCDH7	protocadherin 7	*5099*			**0.8**	
PCK2	phosphoenolpyruvate carboxykinase 2 (mitochondrial)	*5106*	**0.810**			
PECI	peroxisomal D3,D2-enoyl-CoA isomerase	*10455*	**0.730**		**2.38**	
PEX11G	peroxisomal biogenesis factor 11 gamma	*92960*	**1.16**			
PFKFB3	6-phosphofructo-2-kinase/ fructose-2,6-biphosphatase 3	*5209*			**0.740**	**0.890**
PFKFB4	6-phosphofructo-2-kinase/ fructose-2,6-biphosphatase 4	*5210*				**1.12**

PFKP	phosphofructokinase, platelet	*5214*			**0.820**	
PGAM1	phosphoglycerate mutase 1 (brain)	*5223*	**2.21**			
PLA2G16	phospholipase A2, group XVI	*11145*			**1.25**	**0.750**
PLD6	phospholipase D family, member 6	*201164*				**1.08**
PLOD1	procollagen-lysine 1, 2-oxoglutarate 5-dioxygenase 1	*5351*	**2.22**			
PNPLA7	patatin-like phospholipase domain containing 7	*375775*	**1.55**			**1.6**
PPAP2A	phosphatidic acid phosphatase type 2A	*8611*			**1.13**	**0.790**
PPIC	peptidylprolyl isomerase C (cyclophilin C)	*5480*	**0.840**			
PPP1R1B	protein phosphatase 1, regulatory (inhibitor) subunit 1B	*84152*	**0.850**			
PRAC	prostate cancer susceptibility candidate	*84366*			**0.700**	
PRAME	preferentially expressed antigen in melanoma	*23532*			**1.22**	
PRICKLE1	prickle homolog 1 (Drosophila)	*144165*			**0.880**	
PRKACB	protein kinase, cAMP-dependent, catalytic, beta	*5567*	**1.81**			
PROSC	proline synthetase co-transcribed homolog (bacterial)	*11212*			**1.2**	
PRPH	peripherin	*5630*	**1.65**			**1.27**
PRSS8	protease, serine, 8	*5652*	**1.51**			
PRUNE2	prune homolog 2 (Drosophila)	*158471*	**0.28**			
PSAP	prosaposin	*5660*	**1.15**		**0.610**	
PTTG1	pituitary tumor-transforming 1	*9232*			**1.28**	
RAMP1	receptor (G protein-coupled) activity modifying protein 1	*10267*	**3.67**			
RAP1GAP	RAP1 GTPase activating protein	*5909*	**0.740**			**1.73**
RBPMS2	RNA binding protein with multiple splicing 2	*348093*			**1.18**	
RCN1	reticulocalbin 1, EF-hand calcium binding domain	*5954*			**0.770**	
RCOR2	REST corepressor 2	*283248*	**1.12**		**1.42**	**1.04**
RHOBTB3	Rho-related BTB domain containing 3	*22836*	**1.15**			
RHOU	ras homolog gene family, member U	*58480*	**0.440**		**1.2**	**0.78**
RNASE4	ribonuclease, RNase A family, 4	*6038*	**0.78**			
RND3	Rho family GTPase 3	*390*				**0.910**
RNF103	ring finger protein 103	*7844*	**0.78**			
RPL13	ribosomal protein L13	*6137*	**0.790**			
RPL23A	ribosomal protein L23a	*6147*			**0.820**	
RPL29	ribosomal protein L29	*6159*				**0.870**
RTN4	reticulon 4	*57142*	**1.16**			**1.18**

S100P	S100 calcium binding protein P	*6286*	**0.720**		**0.380**	
SBK1	SH3-binding domain kinase 1	*388228*	**1.78**		**0.770**	
SCML1	sex comb on midleg-like 1 (Drosophila)	*6322*				**0.78**
SCNN1A	sodium channel, nonvoltage-gated 1 alpha	*6337*	**1.53**			**0.680**
SEL1L3	sel-1 suppressor of lin-12-like 3 (C. elegans)	*23231*				**1.09**
SEPT5	septin 5	*5413*	**1.77**			**1.25**
SERF1B	small EDRK-rich factor 1B (centromeric)	*728492*	**1.28**			
SERPINB6	serpin peptidase inhibitor, clade B (ovalbumin), member 6	*5269*				**1.35**
SERPINE2	serpin peptidase inhibitor, clade E (nexin, plasminogen activator inhibitor type 1), member 2	*5270*	**0.620**	2.27		**0.870**
SERPINI1	serpin peptidase inhibitor, clade I (neuroserpin), member 1	*5274*				**1.8**
SGCE	sarcoglycan, epsilon	*8910*			**1.21**	
SH3BGRL	SH3 domain binding glutamic acid-rich protein like	*6451*	**1.54**			
SHROOM3	shroom family member 3	*57619*				**0.760**
SLC16A3	solute carrier family 16, member 3 (monocarboxylic acid transporter 4)	*9123*	**1.41**		**1.31**	
SLC25A4	solute carrier family 25 (mitochondrial carrier; adenine nucleotide translocator), member 4	*291*				**0.820**
SLC25A6	solute carrier family 25 (mitochondrial carrier; adenine nucleotide translocator), member 6	*293*	**0.750**		**0.830**	
SLC2A12	solute carrier family 2 (facilitated glucose transporter), member 12	*154091*	**1.28**			
SLC6A10P	solute carrier family 6 (neurotransmitter transporter, creatine), member 10 (pseudogene)	*386757*			**0.820**	**0.850**
SNHG5	small nucleolar RNA host gene 5 (non-protein coding)	*387066*	**0.5**			
SNHG6	small nucleolar RNA host gene 6 (non-protein coding)	*641638*				**1.13**
SNHG7	small nucleolar RNA host gene 7 (non-protein coding)	*84973*	**0.820**			
SNHG8	small nucleolar RNA host gene 8 (non-protein coding)	*100093630*	**0.560**			
SNHG9	small nucleolar RNA host gene 9 (non-protein coding)	*735301*	**1.15**			

SNORA18	small nucleolar RNA, H/ACA box 18	*677805*	**0.660**			
SNRPN	small nuclear ribonucleoprotein polypeptide N	*6638*	**1.53**			
SOX11	SRY (sex determining region Y)-box 11	*6664*			**0.670**	
SPON2	spondin 2, extracellular matrix protein	*10417*	**1.73**			**2.62**
SQSTM1	sequestosome 1	*8878*	**0.590**			**1.22**
SSR2	signal sequence receptor, beta (translocon-associated protein beta)	*6746*	**0.790**			
STEAP1	six transmembrane epithelial antigen of the prostate 1	*26872*	**1.49**			
STK24	serine/threonine kinase 24	*8428*	**1.5**			**1.2**
SYT4	synaptotagmin IV	*6860*	**5.84**			
SYT7	synaptotagmin VII	*9066*	**1.19**			
TACC1	transforming, acidic coiled-coil containing protein 1	*6867*	**6.68**			
TAGLN2	transgelin 2	*8407*				**0.860**
TARP	TCR gamma alternate reading frame protein	*445347*				**4.34**
TBX3	T-box 3	*6926*	**1.29**		**1.35**	
TCEA3	transcription elongation factor A (SII), 3	*6920*	**0.790**			
TCEAL3	transcription elongation factor A (SII)-like 3	*85012*	**5.76**			**1.12**
TFF3	trefoil factor 3 (intestinal)	*7033*	**1.94**			
TGIF1	TGFB-induced factor homeobox 1	*7050*	**0.790**		**0.58**	
THNSL2	threonine synthase-like 2 (S. cerevisiae)	*55258*	**1.25**			
TLE1	transducin-like enhancer of split 1 (E(sp1) homolog, Drosophila)	*7088*	**0.37**			
TM4SF1	transmembrane 4 L six family member 1	*4071*			**0.630**	
TMEM14C	transmembrane protein 14C	*51522*	**0.810**			**1.18**
TMEM158	transmembrane protein 158 (gene/pseudogene)	*25907*	**0.730**			**1.46**
TMEM194A	transmembrane protein 194A	*23306*	**0.820**			
TMEM205	transmembrane protein 205	*374882*	**21.57**			
TMPRSS2	transmembrane protease, serine 2	*7113*			**0.8**	**1.23**
TMSB10	thymosin beta 10	*9168*	**0.860**			
TMSB15A	thymosin beta 15a	*11013*			**0.750**	
TMSB15B	thymosin beta 15B	*286527*			**0.78**	
TNFRSF19	tumor necrosis factor receptor superfamily, member 19	*55504*	**3.37**			
TP53TG3	TP53 target 3	*24150*	**1.65**			
TPD52L1	tumor protein D52-like 1	*7164*	**0.78**			

TRIM68	tripartite motif containing 68	*55128*	**2.18**			
TSC2	tuberous sclerosis 2	*7249*				**0.95**
TSC22D3	TSC22 domain family, member 3	*1831*			**0.610**	
TSGA14	testis specific, 14	*95681*	**1.21**			
TSPAN8	tetraspanin 8	*7103*	**0.21**			
TSPAN9	tetraspanin 9	*10867*	**1.65**			
TSPY2	testis specific protein, Y-linked 2	*64591*	**1.17**			
TSPY8	testis specific protein, Y-linked 8	*728403*	**1.19**			**0.960**
TST	thiosulfate sulfurtransferase (rhodanese)	*7263*	**0.830**			
TUBB3	tubulin, beta 3	*10381*	**1.77**		**0.750**	
TXNDC5	thioredoxin domain containing 5 (endoplasmic reticulum)	*81567*	**0.64**			
VAMP8	vesicle-associated membrane protein 8 (endobrevin)	*8673*	**0.840**			
VCX	variable charge, X-linked	*26609*			**1.75**	
VCX2	variable charge, X-linked 2	*51480*	**2.28**		**1.97**	
VCX3A	variable charge, X-linked 3A	*51481*	**6.23**		**1.72**	
VCX3B	variable charge, X-linked 3B	*425054*			**1.78**	
VCY	variable charge, Y-linked	*9084*	**1.78**		**1.87**	**0.960**
VEGFB	vascular endothelial growth factor B	*7423*	**7.29**			
WBP5	WW domain binding protein 5	*51186*	**1.32**		**0.690**	
WDR72	WD repeat domain 72	*256764*	**3**			
WNT5A	wingless-type MMTV integration site family, member 5A	*7474*				**1.07**
XAGE1A	X antigen family, member 1A	*653219*	**2.04**		**0.820**	
YBX2	Y box binding protein 2	*51087*			**1.55**	
ZDHHC4	zinc finger, DHHC-type containing 4	*55146*	**1.22**		**1.23**	
ZDHHC7	zinc finger, DHHC-type containing 7	*55625*	**1.26**		**0.820**	
ZDHHC8	zinc finger, DHHC-type containing 8	*29801*	**0.8**		**0.750**	
ZFYVE21	zinc finger, FYVE domain containing 21	*79038*				**1.08**
ZIC2	Zic family member 2 (odd-paired homolog, Drosophila)	*7546*	**0.680**			**0.890**
ZNF385B	zinc finger protein 385B	*151126*				**1.24**
ZSCAN18	zinc finger and SCAN domain containing 18	*65982*	**2.43**			
ZYX	zyxin	*7791*	**1.39**			**1.13**

Supplementary Table S4: (In Reference to Figure 6D)

Ingenuity Canonical Pathways	-log (*p*-value)	Ratio	Molecules
Hepatic Fibrosis / Hepatic Stellate Cell Activation	3.51E+0	1.52E-2	BAMBI, COL4A5, IGFBP5
Leucine Degradation I	2.21E+0	1.11E-1	MCCC2
Inhibition of Angiogenesis by TSP1	1.63E+0	2.94E-2	GUCY1A3
Neuroprotective Role of THOP1 in Alzheimer's Disease	1.57E+0	2.50E-2	MME
Phototransduction Pathway	1.44E+0	1.89E-2	GUCY1A3
Antiproliferative Role of Somatostatin Receptor 2	1.37E+0	1.59E-2	GUCY1A3
VDR/RXR Activation	1.28E+0	1.28E-2	IGFBP5
Neuregulin Signaling	1.23E+0	1.14E-2	TMEFF2
IGF-1 Signaling	1.19E+0	1.03E-2	IGFBP5
Nitric Oxide Signaling in the Cardiovascular System	1.18E+0	1.00E-2	GUCY1A3
Corticotropin Releasing Hormone Signaling	1.13E+0	9.01E-3	GUCY1A3
Sperm Motility	1.10E+0	8.26E-3	GUCY1A3
Cellular Effects of Sildenafil (Viagra)	1.07E+0	7.75E-3	GUCY1A3
Relaxin Signaling	1.05E+0	7.41E-3	GUCY1A3
Synaptic Long Term Depression	1.03E+0	7.04E-3	GUCY1A3
eNOS Signaling	1.03E+0	7.04E-3	GUCY1A3
Gap Junction Signaling	9.93E-1	6.45E-3	GUCY1A3
Dopamine-DARPP32 Feedback in cAMP Signaling	9.77E-1	6.21E-3	GUCY1A3
Endothelin-1 Signaling	9.50E-1	5.81E-3	GUCY1A3
Sertoli Cell-Sertoli Cell Junction Signaling	9.36E-1	5.62E-3	GUCY1A3
Glucocorticoid Receptor Signaling	7.60E-1	3.64E-3	ANXA1

Supplementary Table S5: (In Reference to Figure 7D)

Ingenuity Canonical Pathways	-log (*p*-value)	Ratio	Molecules
Gluconeogenesis I	2.62E+0	8.00E-2	ENO2,ME1
Creatine-phosphate Biosynthesis	1.94E+0	2.50E-1	CKB
Galactose Degradation I (Leloir Pathway)	1.84E+0	2.00E-1	GALK1
Phosphatidylcholine Biosynthesis I	1.70E+0	1.43E-1	CHPT1
TR/RXR Activation	1.60E+0	2.35E-2	ME1,SLC16A3
Virus Entry via Endocytic Pathways	1.56E+0	2.25E-2	HLA-A,CXADR
Amyotrophic Lateral Sclerosis Signaling	1.48E+0	2.04E-2	GPX1,PRPH
Choline Biosynthesis III	1.43E+0	7.69E-2	CHPT1
Colanic Acid Building Blocks Biosynthesis	1.40E+0	7.14E-2	GALK1
Parkinson’s Signaling	1.34E+0	6.25E-2	SEPTIN 5
RhoA Signaling	1.31E+0	1.64E-2	LPAR2,SEPT5
Glutathione Redox Reactions I	1.27E+0	5.26E-2	GPX1
Bupropion Degradation	1.15E+0	4.00E-2	CYP2J2
Glycolysis I	1.15E+0	4.00E-2	ENO2
Acetone Degradation I (to Methylglyoxal)	1.14E+0	3.85E-2	CYP2J2
Germ Cell-Sertoli Cell Junction Signaling	1.10E+0	1.25E-2	ZYX,RHOU
Cytotoxic T Lymphocyte-mediated Apoptosis of Target Cells	1.05E+0	3.12E-2	HLA-A
Oncostatin M Signaling	1.03E+0	2.94E-2	MT2A
Interferon Signaling	1.03E+0	2.94E-2	IFITM2
Estrogen Biosynthesis	9.91E-1	2.70E-2	CYP2J2
Antigen Presentation Pathway	9.91E-1	2.70E-2	HLA-A
Neuroprotective Role of THOP1 in Alzheimer’s Disease	9.59E-1	2.50E-2	HLA-A
Integrin Signaling	9.37E-1	9.95E-3	ZYX,RHOU
Role of Oct4 in Mammalian Embryonic Stem Cell Pluripotency	9.02E-1	2.17E-2	CCNF
Graft-versus-Host Disease Signaling	8.85E-1	2.08E-2	HLA-A
Autoimmune Thyroid Disease Signaling	8.76E-1	2.04E-2	HLA-A
Semaphorin Signaling in Neurons	8.45E-1	1.89E-2	RHOU
Nicotine Degradation III	8.37E-1	1.85E-2	CYP2J2
Signaling by Rho Family GTPases	8.30E-1	8.55E-3	RHOU,SEPT5
Actin Nucleation by ARP-WASP Complex	8.23E-1	1.79E-2	RHOU
Glioma Invasiveness Signaling	8.16E-1	1.75E-2	RHOU
Melatonin Degradation I	8.16E-1	1.75E-2	CYP2J2
Induction of Apoptosis by HIV1	7.95E-1	1.67E-2	SLC25A6
Superpathway of Melatonin Degradation	7.82E-1	1.61E-2	CYP2J2
Nicotine Degradation II	7.76E-1	1.59E-2	CYP2J2
Remodeling of Epithelial Adherens Junctions	7.46E-1	1.47E-2	ZYX
Caveolar-mediated Endocytosis Signaling	7.29E-1	1.41E-2	HLA-A
T Helper Cell Differentiation	7.29E-1	1.41E-2	BCL6
Allograft Rejection Signaling	6.54E-1	1.16E-2	HLA-A
TGF-Î² Signaling	6.50E-1	1.15E-2	TGIF1
CTLA4 Signaling in Cytotoxic T Lymphocytes	6.46E-1	1.14E-2	HLA-A
Crosstalk between Dendritic Cells and Natural Killer Cells	6.41E-1	1.12E-2	HLA-A
OX40 Signaling Pathway	6.41E-1	1.12E-2	HLA-A

Regulation of Actin-based Motility by Rho	6.33E-1	1.10E-2	RHOU
Communication between Innate and Adaptive Immune Cells	6.33E-1	1.10E-2	HLA-A
VEGF Signaling	6.29E-1	1.09E-2	EIF2S3
p53 Signaling	6.05E-1	1.02E-2	SERPINE2
Cholecystokinin/Gastrin-mediated Signaling	5.93E-1	9.90E-3	RHOU
Sphingosine-1-phosphate Signaling	5.65E-1	9.17E-3	RHOU
Type I Diabetes Mellitus Signaling	5.62E-1	9.09E-3	HLA-A
phagosome formation	5.58E-1	9.01E-3	RHOU
GÎ±12/13 Signaling	5.39E-1	8.55E-3	LPAR2
HMGB1 Signaling	5.30E-1	8.33E-3	RHOU
phagosome maturation	5.30E-1	8.33E-3	HLA-A
Adipogenesis pathway	5.09E-1	7.87E-3	BSCL2
eNOS Signaling	4.70E-1	7.04E-3	LPAR2
Regulation of eIF4 and p70S6K Signaling	4.60E-1	6.85E-3	EIF2S3
Glioblastoma Multiforme Signaling	4.60E-1	6.85E-3	RHOU
Epithelial Adherens Junction Signaling	4.60E-1	6.85E-3	ZYX
GÎ±q Signaling	4.57E-1	6.80E-3	RHOU
AMPK Signaling	4.55E-1	6.76E-3	PFKFB3
CXCR4 Signaling	4.46E-1	6.58E-3	RHOU
Axonal Guidance Signaling	4.45E-1	4.62E-3	RTN4,ADAMTS1
Tec Kinase Signaling	4.32E-1	6.33E-3	RHOU
Cdc42 Signaling	4.14E-1	5.99E-3	HLA-A
RhoGDI Signaling	4.02E-1	5.78E-3	RHOU
B Cell Receptor Signaling	3.96E-1	5.68E-3	BCL6
Dendritic Cell Maturation	3.90E-1	5.59E-3	HLA-A
Production of Nitric Oxide and Reactive Oxygen Species in Macrophages	3.88E-1	5.56E-3	RHOU
IL-8 Signaling	3.81E-1	5.43E-3	RHOU
EIF2 Signaling	3.79E-1	5.41E-3	EIF2S3
ILK Signaling	3.78E-1	5.38E-3	RHOU
mTOR Signaling	3.74E-1	5.32E-3	RHOU
Thrombin Signaling	3.69E-1	5.24E-3	RHOU
Systemic Lupus Erythematosus Signaling	3.24E-1	4.55E-3	HLA-A
Cardiac Hypertrophy Signaling	3.20E-1	4.48E-3	RHOU
Huntington’s Disease Signaling	3.10E-1	4.35E-3	RCOR2
Colorectal Cancer Metastasis Signaling	3.02E-1	4.24E-3	RHOU
Phospholipase C Signaling	2.98E-1	4.18E-3	RHOU
Protein Ubiquitination Pathway	2.79E-1	3.92E-3	HLA-A

Supplementary Table S6: PCR Primers

Gene	Forward Primer	Reverse Primer
AR (Exon 1–2)	ATCCCAGTCCCACTTGTGTC	GGTCTTCTGGGTGGAAAGT
AR (Exon 4)	CGGAAGCTGAAGAAACTTGG	ATGGCTTCCAGGACATTCAG
AR V7	CCATCTTGTCGTCTTCGGAAATGTTATGA	TTTGAATGAGGCAAGTCAGCCTTTCT
GAPDH	GAGTCAACGGATTTGGTCGT	TTGATTTTGGAGGGATCTCG
PSA (*KLK3*)	TCATCCTGTCTCGGATTGTG	ATATCGTAGAGCGGGTGTGG
TMPRSS2	TGTGGTCCCTTCCAATGCTGTG	TGCTCATGGTTATGGCACTTGGC
